# Use of Digital Technology for Developing Communication Skills in Undergraduate and Postgraduate Medical Education: Scoping Review

**DOI:** 10.2196/87012

**Published:** 2026-04-20

**Authors:** Princella Seripenah, Heidi Emery, Bakula Patel, Edward Tyrrell, Julie Carson, Jo Leonardi-Bee, Catrin Evans, Emma Wilson, Jaspal Taggar

**Affiliations:** 1 Primary Care Education Unit, Centre for Academic Primary Care, School of Medicine, University of Nottingham, Room C37, C Floor, The Medical School, Queens Medical Centre, Nottingham, NG7 2UH, United Kingdom, +44 115 82 30445; 2 Education Centre, School of Medicine, University of Nottingham, Nottingham, United Kingdom; 3 Centre for Public Health and Epidemiology, School of Medicine, University of Nottingham, Nottingham, United Kingdom; 4 Centre for Evidence Based Healthcare, Faculty of Medicine and Health Sciences, University of Nottingham, Nottingham, United Kingdom

**Keywords:** digital technology, communication skills, medical education, undergraduate training, postgraduate training, doctor–patient communication, virtual simulation, video-based learning, educational technology, scoping review.

## Abstract

**Background:**

Effective doctor-patient communication is fundamental to safe, high-quality health care and is a core competency across undergraduate and postgraduate medical education. Communication skills training (CST) has traditionally relied on workforce-intensive methods such as role-play and standardized patient encounters, which face increasing pressure from rising student numbers, constrained faculty capacity, and growing clinical workloads. Digital technologies offer scalable, flexible alternatives, yet the extent, educational design, and strength of evidence supporting digital CST remain unclear.

**Objective:**

This study aimed to comprehensively map the digital technologies used for CST in undergraduate and postgraduate medical education, examine the reported outcomes in the context of educational theory, and identify gaps relevant for future research and clinical practice.

**Methods:**

This scoping review followed Joanna Briggs Institute (JBI) methodology and is reported in accordance with PRISMA-ScR (Preferred Reporting Items for Systematic Reviews and Meta-Analyses Extension for Scoping Reviews) guidelines. Four electronic databases (Medline, Embase, CINAHL, ERIC) were searched from inception to January 5, 2026. Eligible studies examined any digital technology used to support active, 2-way CST for undergraduate or postgraduate medical learners. Passive learning approaches were excluded. Data were synthesized descriptively. To support structured interpretation of heterogeneous outcomes, interventions were mapped to Kolb’s experiential learning cycle to examine learning processes and to Kirkpatrick’s evaluation model to assess depth of educational and translational impact.

**Results:**

A total of 11,179 records were identified, of which 121 studies met the inclusion criteria. Most studies were published within the past decade (92/121, 76%) and were conducted in North America and Europe (93/121, 76.9%), with 58.7% (71/121) of studies focusing on undergraduate learners. Recording-based tools (51/121, 41.8%), live stream platforms (33/121, 27%), and virtual patient simulators (32/121, 26.2%) were the most used digital technologies. General communication and history taking was the most frequent topic taught. Only 28.1% (34/121) of studies used validated objective outcome measures. Educationally, digital interventions overwhelmingly supported early stages of experiential learning (120/121, 99.2%), with almost no progression to abstract conceptualization or active experimentation. Outcome evaluation was similarly limited in depth; most studies assessed outcomes at Kirkpatrick Levels 1 and 2. Few studies evaluated behavior change in clinical practice (6/121, 5%) or patient-level outcomes (1/121, 0.8%). A small but growing subset of studies incorporated artificial intelligence, primarily within virtual patient simulators, showing promising but methodologically limited evidence.

**Conclusions:**

Although digital CST interventions show promise for supporting early-stage learning outcomes, the evidence is constrained by weak study designs, inconsistent use of validated measures, and minimal real-world evaluation. Current technologies support only initial phases of experiential learning, with no evidence of progression to competency development or translation into improved patient care. For educators investing in digital CST, these technologies should be integrated thoughtfully within broader curricula rather than treated as standalone solutions, accompanied by evaluation extending to clinical outcomes. Future research that prioritizes robust comparative designs evaluating whether digital training meaningfully improves clinical communication and patient care is warranted.

## Introduction

### Background

Effective doctor-patient communication is fundamental to the delivery of safe and high-quality care. Strong communication enhances patient outcomes by building trust, collaborative decision-making, improved treatment adherence, and better self-care [1]. This importance is reflected in empirical evidence showing that poorer physician communication is associated with higher risks of patient nonadherence, while communication skills training (CST) is linked to meaningful improvements in adherence and other patient-relevant outcomes [2]. Furthermore, interpersonal communication is a core graduate competency for medical training programs globally, and the need to develop and refine communication skills throughout a medical career is recognized as a critical professional standard [3,4]. However, many communication skills are not acquired spontaneously during medical training and require training for their development [5].

Methods of teaching communication skills during medical education have traditionally relied on didactic lectures, role-playing, and standardized patient interactions facilitated by more senior clinicians [6]. These are workforce-intensive methods, particularly when personalized, specific feedback is provided, which has been shown to be effective in improving skill acquisition [6]. Pressures on effective communication skills teaching are further compounded by increasing medical student numbers [7], global shortage of health professionals [8,9], and rising clinical workloads among supervisors [10].

Digital technologies have therefore been increasingly adopted in medical education as a means of addressing these structural constraints. Unlike traditional communication skills teaching, which depends on synchronous, staff-intensive delivery, digital approaches such as virtual patient (VP) simulators and online modules require substantial upfront development but can subsequently be reused at scale, offer flexible and repeated access, and enable learners to practice a wider range of communication scenarios than is feasible in conventional formats [11-13]. These approaches support self-directed learning and allow simulation of interactions that are difficult to stage with standardized patients, including communication involving rare or complex clinical presentations. As a result, digital technologies are increasingly viewed as a means of expanding training capacity and equity while reducing reliance on faculty-intensive delivery and preserving patient safety [11].

There has been a rapid acceleration in the use of digital technology within medical education since 2018, particularly following the shift toward virtual learning environments during the COVID-19 pandemic [14,15], though its application specifically for teaching and learning communication skills remains less well evidenced. Emerging digital modalities, including immersive virtual reality and artificial intelligence (AI)–supported interventions, are increasingly being explored for CST, reflecting a widening technological landscape beyond conventional online or recording-based approaches [16]. Previous reviews examining digital approaches to CST in medical education have tended to adopt narrow foci. For example, Kyaw et al [11] restricted inclusion to randomized controlled trials and focused primarily on knowledge and skills outcomes without examining learning processes. Similarly, Fernández-Alcántara et al [17] concentrated exclusively on virtual simulation tools. Reviews of emerging technologies such as AI [18] have similarly focused on specific modalities rather than examining outcomes across the broader landscape of digital approaches. Of particular importance in the context of clinical education, no previous research has systematically examined how digital interventions align with established educational learning theories or assessed whether evaluation extends beyond educational settings to measure impact on clinical practice.

Educational theory provides a structured lens for interpreting heterogeneous outcomes across diverse digital interventions, enabling appraisal not only of whether learning occurs but how learning is supported and whether it progresses beyond individual knowledge acquisition toward behavioral and practice-level impact [19]. Without such theoretical grounding, the evidence base remains fragmented by technology type rather than organized around learning mechanisms and translational relevance. This scoping review is informed by two well-established theoretical frameworks in medical education that have not previously been integrated within CST. Kolb’s experiential learning cycle [20] is particularly relevant to CST as it captures how learners develop communication competencies through concrete experience, reflection, conceptualization, and experimentation. Kirkpatrick’s evaluation model [21] complements this by providing a framework to assess the depth and real-world impact of interventions, from learner reactions through to practice-level outcomes. Together, these frameworks provide a dual-lens approach that bridges individual learning processes with clinical translation.

### Research Aim

This scoping review mapped digital technologies used to develop clinical communication competencies in undergraduate and postgraduate medical education and examined reported outcomes through a dual lens of pedagogical theories for clinical education, notably experiential learning and translational impact.

### Research Objectives

Our overarching aim was achieved by the following objectives:

1. To characterize digital technologies used for developing communication skills in undergraduate and postgraduate medical education, including technology modalities, their educational context, learner outcomes, and how their use has evolved over time
2. To examine how digital communication skills interventions align with experiential learning processes and the depth of educational impact, as mapped using Kolb’s experiential learning cycle and Kirkpatrick’s evaluation model
3. To identify evidence gaps in the design, evaluation, and reporting of digital communication skills interventions in relation to behavioral change and clinical practice outcomes

## Methods

### Study Design

This scoping review was conducted using the Joanna Briggs Institute (JBI) methodological framework [22] and reported adhering to the PRISMA-ScR (Preferred Reporting Items for Systematic Reviews and Meta-Analyses Extension for Scoping Reviews) [23]. The PRISMA-ScR checklist is included in Checklist 1. This protocol was prospectively registered with Open Science Framework [24].

### Research Question

We defined our research question as “What is known in the existing literature about the use of digital technologies for CST in undergraduate and postgraduate medical education, and to what extent do reported outcomes reflect experiential learning processes and extend beyond individual learning toward behavioral change and clinical practice-level impact?”

### Eligibility Criteria

Studies were included if they met the criteria outlined in the following sections.

#### Participants

Participants were learners participating in medical education. For the purposes of this review, we referred to learners as either undergraduates or postgraduates.

Undergraduates were defined as medical students at the preregistration level (ie, individuals enrolled in a medical degree program who have not yet obtained a license or registration to practice).

Postgraduates were defined as medical doctors at the postregistration level, including those in internship, residency, specialty training, or other forms of postgraduate education and continuing professional development.

#### Concept

The concept involved the delivery of CST through digital and technology-enhanced methods. Communication skills included content (what is said), process (how the communication is done), and perceptual (underlying thoughts, emotions, and clinical reasoning) skills as described by Kurtz et al [25]. For this review, we further defined communication skills as the capacity for at least 2-way, dynamic interaction between the learner and a patient (or patient’s representative) to convey health care information between the parties involved. Passive interactions, such as watching a premade video, were excluded.

For the purposes of this review, a patient was defined as either a real human patient, a trained actor (for example, a standardized patient), or a digital or simulated representation designed to support the communication of health care information. Digital technologies were defined as digital systems, tools, or platform devices that use electronic data processing to support the delivery, practice, or assessment of communication skills through active learner engagement, including interactive, simulated, or feedback-enabled educational activities. Studies describing only the assessment of communication skills (eg, in Objective Structured Clinical Examination situations) were excluded.

#### Context

The context included any setting where communication skills were taught, including academic institutions, clinical environments, and synchronous or asynchronous simulation-based scenarios. The interactions were either real-world or role-play encounters.

#### Evidence Types

All primary study designs were included to gain comprehensive evidence of the topic. We excluded secondary sources such as systematic reviews, scoping reviews, and meta-analyses, as well as nonresearch publications including study protocols, expert opinion, discussion papers, letters, comments, editorials, and book chapters. Conference abstracts without accompanying full-text publications were excluded due to insufficient detail for data extraction. Primary studies cited within relevant reviews were extracted and assessed for eligibility.

### Search Strategy

An initial scoping search in Medline (OVID) identified relevant index terms and keywords, which informed the development of a comprehensive search strategy. The strategy incorporated both keywords and controlled vocabulary across three core concept areas: medical education (undergraduate and postgraduate), digital technologies (VPs, simulation, online learning, AI, virtual reality), and communication skills, combined using Boolean operators. The search strategy was adapted from a previously published scoping review on digital education and communication skills [11] and refined to align with this review’s inclusion criteria. During development, the strategy was reviewed in consultation with a research librarian and refined following feedback from the review team, including methodological experts. The final strategy was tested iteratively in Medline and adapted across all selected databases (see Multimedia Appendix 1). Reporting of the search strategy follows the PRISMA-S (Preferred Reporting Items for Systematic Reviews and Meta-Analyses Extension for Literature Searches) checklist [26] (Checklist 2).

The electronic databases Medline (via Ovid), Embase (via Ovid), Educational Resource Information Center (ERIC; via EBSCO), and Cumulative Index to Nursing and Allied Health Literature (CINAHL; via EBSCO) were searched from inception to January 5, 2026. Searches were initially conducted in November 2024, re-run in August 2025, and subsequently updated on January 5, 2026, using the same search strategy to identify newly published studies. No changes were made to the search terms or eligibility criteria across the three search runs. Reference lists of included articles and relevant reviews were hand-searched for additional studies.

We imposed no language restrictions on any of the searches. Non-English texts were translated using DeepL Translator software [27]. A standardized quality assessment tool was not used in this review, as the primary aim was to map existing research rather than conduct a detailed critical assessment of individual studies.

### Selection of Sources of Evidence

Identified studies were uploaded to Covidence [28] and automatically deduplicated. All titles and abstracts were screened independently by two reviewers (HE, PS, or ET), with discrepancies resolved by group discussion with a fourth reviewer (JT). Full texts of potentially eligible studies were screened independently by two reviewers (HE, PS, JC, or BP) with discrepancies resolved through discussion with the whole research group.

### Data Charting Process

Data on study characteristics, learner population and training context, digital technology modality and intervention design features, communication domains addressed, outcome measures and effect direction, use of educational theory, and authors’ conclusions were extracted into a piloted spreadsheet (Multimedia Appendix 2). Data from 10% of included studies were extracted independently by two reviewers (HE, PS, ET, or BP), with findings compared and discussed with the research group. After a high level of agreement was achieved, remaining studies were extracted by one reviewer (HE or PS).

### Synthesis of Results

A descriptive analysis was conducted and reported using frequencies and percentages. Studies were categorized by their primary mode of delivery and interaction for developing communication. To support structured synthesis of heterogeneous outcomes, we applied two complementary frameworks:

1. *Kolb’s experiential learning cycle* [20]: Interventions were assessed to identify which stages of experiential learning were explicitly supported (where interventions supported more than one stage of the cycle, all relevant stages were recorded):
  - Stage 1 (concrete experience): direct patient/scenario interaction
  - Stage 2 (reflective observation): guided reflection on experiences
  - Stage 3 (abstract conceptualization): synthesis of principles from experiences
  - Stage 4 (active experimentation): application of learning in new contexts
2. *Kirkpatrick’s evaluation model* [21]: Study outcomes were mapped to increasing levels of evaluation:
  - Level 1: learner satisfaction or reactions only
  - Level 2: improvement in knowledge or communication skills acquisition
  - Level 3: behavior change in clinical practice
  - Level 4: patient or organizational outcomes

Kirkpatrick’s evaluation model was selected because it is widely recognized in medical education research and enables comparison of outcome depth across diverse intervention types and study designs [29].

The use of these two frameworks allowed consistent appraisal of how interventions were pedagogically structured to support learning processes as well as the depth of outcome assessment. As neither framework was explicitly referenced in included studies, classifications were assigned by authors. Two reviewers (PS and HE) independently conducted all framework assignments, with discrepancies resolved through discussion. This approach enabled systematic comparison across technologies and identification of patterns and evidence gaps in learning processes and outcome depth.

## Results

### Selection of Sources of Evidence

**Figure 1.**
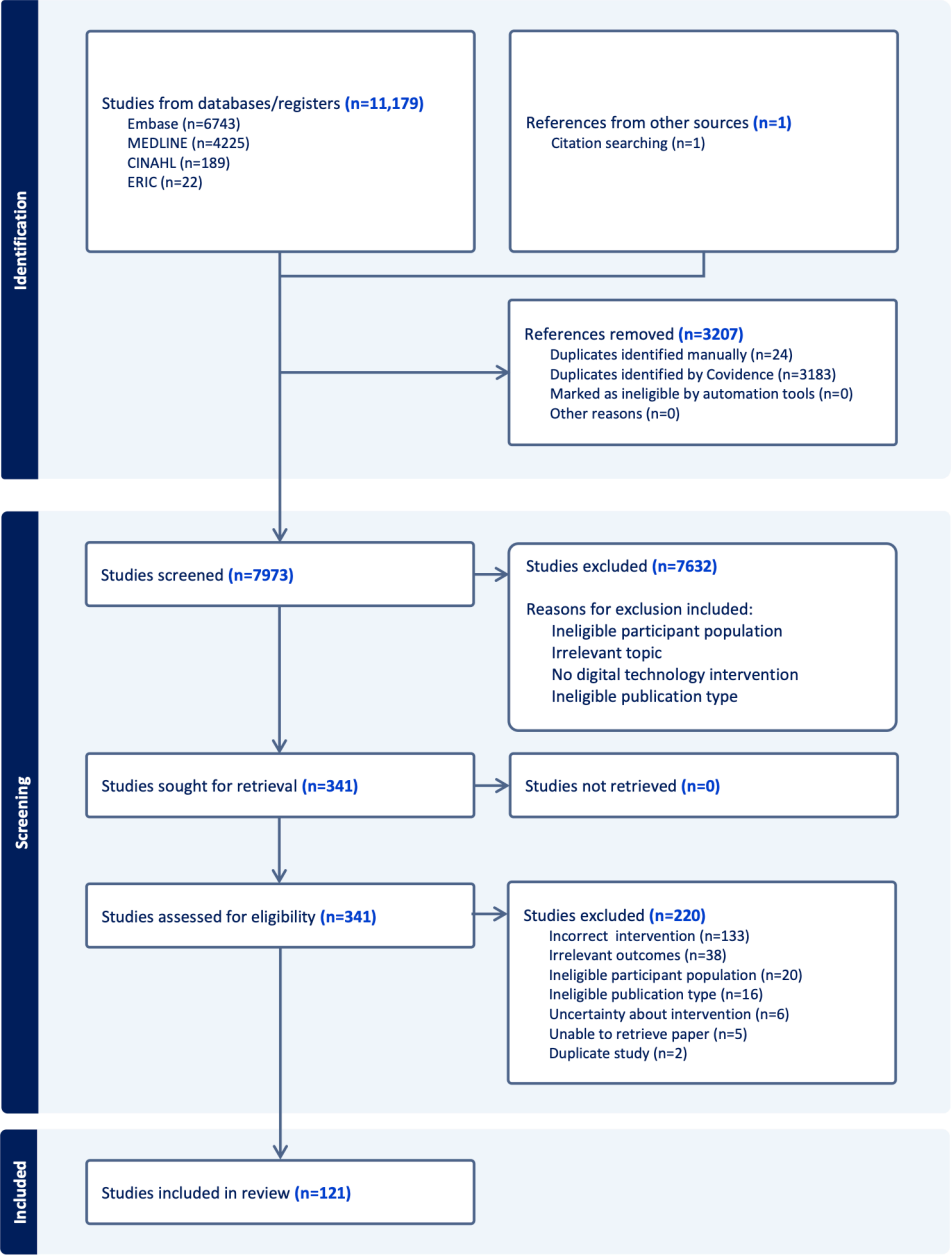
PRISMA (Preferred Reporting Items for Systematic Reviews and Meta-Analyses) flow diagram showing the identification, screening, eligibility, and inclusion of studies using digital technology for developing communication skills in undergraduate and postgraduate medical education.

A total of 11,179 records were identified through database searches, and 1 study was identified via citation searching. Following the removal of 3207 duplicate records, 7973 records were screened. Of these, 341 full-text articles were sought for retrieval, with 336 successfully obtained. After full-text review, 220 studies were excluded based on the predefined eligibility criteria. The reasons for exclusion were incorrect intervention (n=133), irrelevant outcomes (n=38), ineligible participant population (n=19), unsuitable article format (n=16), uncertainty regarding the intervention (n=6), inability to retrieve paper (n=5), and duplicate study (n=1). A total of 121 studies were included in the final synthesis, as detailed in the PRISMA (Preferred Reporting Items for Systematic Reviews and Meta-Analyses) flow diagram (Figure 1). A summary of all included studies is provided in Multimedia Appendix 3 [30-150].

### Characteristics of Sources of Evidence

**Figure 2.**
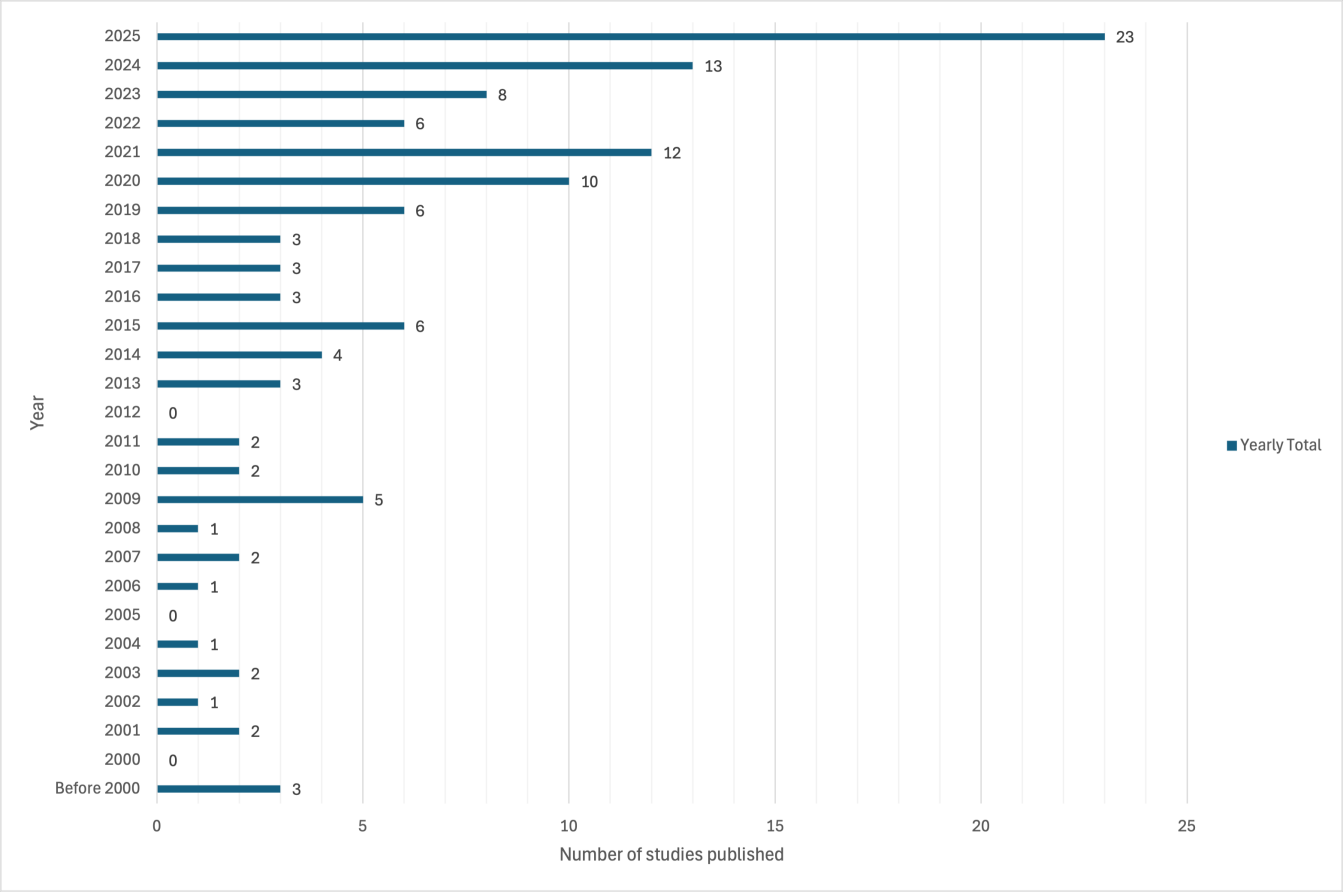
Distribution of studies examining digital communication skills training in medical education by year of publication, illustrating yearly publication trends in digital communication skills training research.

Table 1 summarizes key characteristics of the included studies. The earliest studies were published prior to 2000, with relatively low publication activity until the mid-2010s. Most studies were published between 2015 and 2025 (92/121, 76.0%). Publication output peaked in 2025 (24/121), with high publication activity also noted in 2020, 2021, and 2023. Geographically, most studies originated from North America (48/121, 39.7%) and Europe (45/121, 37.2%), with more limited representation from Asia (16/121, 13.2%), Oceania (7/121, 5.8%), South America (4/121, 3.3%), and Africa (1/121, 0.8%; Figures2  3).

**Table 1.** Characteristics of studies included within the scoping review examining digital communication skills training in medical education (n=121).

Domain and feature	Values, n (%)
Study design	
Quasi-experimental	51 (42.1)
Cross-sectional study	29 (24)
Nonrandomized comparative study	10 (8.3)
Mixed methods	8 (6.6)
Qualitative research	2 (1.7)
Descriptive case study	1 (0.8)
Randomized controlled trial	20 (16.5)
Location (by region)	
North America	48 (39.7)
Europe	45 (37.2)
Asia	16 (13.2)
Oceania	7 (5.8)
South America	4 (3.3)
Africa	1 (0.8)
Number of students	
0‐50	60 (49.6)
51‐100	28 (231)
>100	31 (25.6)
Not reported	2 (1.7)
Learner level	
Undergraduate	71 (58.7)
Postgraduate	45 (37.2)
Mixed	5 (4.1)
Communication skills focus	
General communication and history taking	62 (51.2)
Difficult conversations (BBN^a^, GOC^b^, DOME^c^)	27 (22.3)
Communication with defined patient groups	15 (12.4)
Shared decision-making and consent	7 (5.8)
Telecommunication/remote consultations	5 (4.1)
Patient education and information giving	5 (4.1)

a BBN: breaking bad news.

b GOC: goals of care.

c DOME: disclosure of medical error.

**Figure 3.**
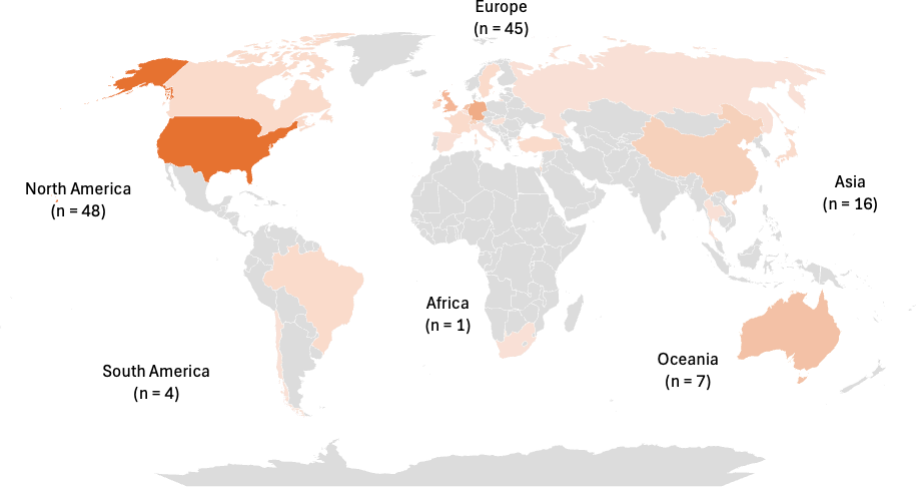
Geographic distribution of studies examining digital communication skills training in medical education by global region.

Quasi-experimental designs were most common (51/121, 42.1%), followed by cross-sectional studies (29/121, 24%) and randomized controlled trials (20/121, 16.5%), with few using cohort, case study, or qualitative methods. Almost one-half of the studies (60/121, 49.6%) involved fewer than 50 students, while the remainder were split between those with 51‐100 students (28/122, 23.1%) and those with more than 100 (31/121, 25.6%). Most studies focused on undergraduate learners (71/121, 58.7%), with a smaller proportion targeting postgraduates (45/121, 37.2%), and a few included mixed groups (5/121, 4.1%).

In terms of CST, general communication and history taking was the most taught (62/121, 51.2%), followed by difficult conversations, such as breaking bad news, goals of care, or disclosure of medical errors (27/121, 22.3%). A smaller proportion of studies focused on communication with defined patient groups (15/121, 12.4%), including adolescents, children, patients with hearing loss or on dialysis, and individuals receiving psychiatric care. Other areas addressed less frequently were shared decision-making and consent (7/121, 5.8%), telecommunication or remote consultations (5/121, 4.1%), and patient education and information giving (5/121, 4.1%).

### Synthesis of Results

#### Digital Technologies Used

##### Overview

Using an inductive approach, 3 main approaches to digital communication training were identified: recording-based tools where audio or video was used for teaching, with reflection and feedback [30-80]; live stream platforms that supported synchronous interaction between learners, educators, or simulated patients [81-113]; and VP simulators encompassing both scripted systems and those driven by AI. “Other” digital tools were investigated by 5 further studies [146-150].

Recording-based tools (51/121, 41.8%) mostly used video recording (42/121, 34.7%), with fewer using audio recording (3/121, 2.5%) or the recording functions of videoconferencing platforms (6/121, 5.0%). These typically involved capturing simulated or real consultations for later review, often followed by structured feedback and reflection.

Live stream platforms (33/121, 27.3%) were classified separately and referred exclusively to real-time videoconferencing without recording, typically involving simulated patient encounters, role-play sessions, or workshops with immediate feedback.

VP simulators (32/121, 26.4%) used computer-based or AI-enabled VP to replicate clinical interactions, sometimes in immersive or branching scenarios that allowed repeated practice.

A further 5 studies (5/121, 4.1%) evaluated other digital tools, including AI-assisted case vignettes, patient portals, translation apps, general communication software, and specialty-specific video case platforms.

##### Recording-Based Digital Tools

The most used approach across all learner groups were recording-based tools [30-80]. These were used fairly equally with postgraduates (26/51, 51%) and undergraduate learners (24/51, 47.1%), demonstrating applicability across training levels, though mixed learner groups were rarely studied (1/51, 2%). Study content was heavily concentrated on general communication and history taking (29/51, 56.9%), followed by difficult conversations such as breaking bad news (11/51, 21.6%). Very few studies addressed communication with defined patient groups (6/51, 11.8%), shared decision-making and consent (4/51, 7.8%), or patient education and information giving (1/51, 2%). The use of these technologies was predominantly beneficial (33/51, 64.7%) [32,34-36,38,41,43-45,48-53,55,56,60-62,64-68,71-73,75-77,79,80], with reported improvements noted in learner performance, reflective capacity, and confidence. However, a notable minority of studies were inconclusive (8/51, 15.7%) [30,33,39,42,54,57,59,63], reported mixed findings (5/51, 9.8%) [31,46,47,74,78], or showed no effect (5/51, 9.8%) [37,40,58,69,70].

##### Live Stream Platforms

Live stream–based interventions were evaluated in 33 studies [81-113]. These were used more frequently with undergraduate learners (20/33, 60.6%) than postgraduates (12/33, 36.4%), with mixed learner groups rarely studied (1/33, 3%). Study content showed greater diversity: general communication and history taking: 11/33, 33.3%; difficult conversations: 8/33, 24.2%; telecommunication or remote consultations: 5/33, 15.2%; communication with defined population groups: 5/33, 15.2%; shared decision-making and consent: 2/33, 6.1%; and patient education and information giving: 2/33, 6.1%. The use of live-streaming platforms was highly beneficial, with more than two-thirds of studies (23/33, 64.9%) demonstrating positive effects including increased communication confidence [82,85,89,95,101-103,105,108,110], improved self-perceived competence [97,98,100,106,109], high learner satisfaction [86-88,90], and, in some cases, objective gains in communication performance [83,84,91,102,104]. However, a notable proportion reported mixed findings (7/33, 21.2%) [81,92-94,96,111,112], while few studies were inconclusive (1/33, 3.0%) [99], were equivalent to traditional teaching methods (1/33, 3.0%) [113], or demonstrated no measurable effect (1/33, 3.0%) [107].

##### VP Simulators

VP simulators were evaluated in 32 studies [114-145]. These were used predominantly with undergraduate learners (24/32, 75%), with fewer at the postgraduate level (5/32, 15.6%) or involving mixed learner groups (3/32, 9.4%). Study content returned a concentrated focus similar to recording-based tools, most often addressing general communication and history taking (19/32, 59.4%), followed by difficult conversations (7/32, 21.9%), with minimal attention to communication with defined patient groups (3/32, 9.4%), patient education (2/32, 6.3%), and shared decision-making and consent (1/32, 3.1%). Reported outcomes were generally positive; nearly two-thirds of studies (21/32, 65.6%) found use of VP simulators beneficial [114,119,120,124-127,129,130,132,134-141,143-145], including improvements in communication performance, empathy, and confidence. Notably, 3 of the 32 studies (9.4%) [133,142,151] reported outcomes equivalent to traditional methods. The remaining studies were inconclusive (4/32, 2.5%) [115,116,118,123], reported mixed findings (2/32, 6.3%) [122,131], or showed no effect (2/32, 6.3%) [117,128].

##### Other Approaches

Tools that did not align with the 3 main categories were evaluated in 5 studies [146-150]. These included AI-assisted case vignettes (1/5, 20%) [146], patient portals for drafting messages (1/5, 20%) [147], speech-to-speech translation apps (1/5, 20%) [148], general communication software (1/5, 20%) [149], and specialty-specific video case platforms (1/5, 20%) [150]. Learners comprised both undergraduates (3/5, 60%) and postgraduates (2/5, 40%). The majority demonstrated improvements (3/5, 60%) in communication practice and confidence [146,147,150]; 1 study was inconclusive (1/5, 20%) [148], and another reported mixed effects (1/5, 20%) [149].

### Trends in the Use of Digital Technologies for CST

Across all learner levels, recording-based approaches were present from the earliest period captured within this review (before 2000) and remained the dominant approach until approximately 2020. Live-streaming platforms expanded markedly after 2020, likely accelerated by the COVID-19 pandemic, with publication numbers surging from sporadic use to 6 studies in 2021 and remaining elevated at 7 studies in 2025. Most strikingly, VP simulators demonstrated explosive recent growth, particularly in 2024 with 14 published studies, representing a dramatic shift in the field’s technological focus (Figure S4 in Multimedia Appendix 4).

These trends varied by learner level. In undergraduate education, the surge in VP simulators was particularly pronounced, with 10 studies published in 2024 alone, while live streaming also showed sustained growth from 2021 onward, plateauing at 3 studies annually by 2025 (Figure S5 in Multimedia Appendix 4). In postgraduate education, the pattern differed notably: Recording-based approaches dominated until the mid-2010s before declining to near absence by 2025, while live streaming showed steady growth after 2020, peaking at 4 studies in 2021‐2022 and 2025 (Figure S6 in Multimedia Appendix 4). VP simulators remained less prominent at the postgraduate level compared with undergraduate settings, showing only gradual uptake in recent years without the dramatic spike observed in undergraduate training.

### Training Assessment Tools Reported

Assessment tools for measuring impact of digital technology on CST varied widely across studies (see Table 2 and Figure S7 in Multimedia Appendix 5). Outcome measures were categorized as either objective or self-reported and further classified by validation status (validated, nonvalidated, or unknown). Classification was based on the type and clarity of evidence regarding communication skill development.

**Table 2.** Assessment tools used by studies examining digital communication skills training in medical education to evaluate outcomes, stratified by learner level and validation status.

Learner level and assessment type		Validated, n (%)^a^	Nonvalidated, n (%)^a^	Unknown, n (%)^a^
Undergraduate (n=71)				
Objective (n=39)		16 (41)	16 (41)	7 (17.9)
Self-reported (n=32)		0 (0)	32 (100)	0 (0)
Postgraduate (n=45)				
Objective (n=31)		16 (51.6)	7 (22.6)	8 (25.8)
Self-reported (n=14)		0 (0)	13 (92.9)	1 (7.1)
Mixed (n=5)				
Objective (n=2)		2 (100)	0 (0)	0 (0)
Self-reported (n=3)		1 (33.3)	2 (66.7)	0 (0)

a Percentages represent the proportion of each validation category within the respective assessment type (objective or self-reported) for each learner level.

Assessment patterns varied by learner level. Among undergraduate learners, objective measures were used slightly more frequently (39/71, 54.9%) than self-reported assessments (32/71, 45.1%). In contrast, postgraduate studies showed a clearer preference for objective assessment (31/45, 68.9%) compared with self-reported tools (14/45, 31.1%). Across all studies, a significant validation gap emerged: Most studies used nonvalidated assessment tools (71/121, 58.7%), while fewer used validated measures (35/121, 28.9%) and a minority having unknown validation status (16/121, 13.2%).

Only 34 of the 121 studies (28.1%) assessed outcomes with a validated objective measure. The objective measures commonly included global rating scales, structured checklists, and simulated patient assessments. A subset of studies used validated frameworks, such as the Calgary-Cambridge Observation Guide, Communication Assessment Tool, SEGUE framework, SPIKES protocol, and the Jefferson Scale of Physician Empathy. Several studies also incorporated qualitative methods such as thematic analysis of interviews or open-ended feedback responses.

### Reported Impact of Digital Technology in Relation to Presence of Comparator Group

Study outcomes were grouped into 5 categories: beneficial, mixed, equivalent, inconclusive, or no effect. Studies were classified as beneficial if they included either objective measures (eg, assessor ratings, checklist scores) or self-reported reflections that indicated improvement or effective demonstration of communication skills. Studies were classified as mixed if they reported improvement in certain aspects of communication skills while showing no improvement or even decline in others. Studies were considered equivalent if they compared a digital intervention to another method (eg, traditional teaching or an alternative digital tool) and found no significant difference in communication skill outcomes between groups. Studies were considered inconclusive if they lacked sufficient outcome data to assess impact on communication skills. Finally, studies were labeled no effect if they explicitly reported no improvement in communication performance postintervention.

Most studies demonstrating positive effects of digital technology for CST (80/121) lacked robust methodological designs. Nearly three-quarters (60/81, 74.1%) used either within-group designs (39/80, 48.8%) or had no comparator at all (20/80, 25%), limiting causal inference. Fewer studies (21/80, 26.2%) included an external comparison group, most often comparing against traditional teaching (11/80, 13.8%), followed by no-intervention controls (4/80, 5%), alternative digital tools (3/80, 3.8%), and other nontraditional teaching methods (3/80, 3.8%).

The use of validated assessment tools further weakened the evidence base: Only 26 of the 80 positive studies (32.5%) used validated measures. Among these 26 validated studies, one-half used within-group designs (13/26, 50%), with smaller proportions comparing against traditional teaching (5/26, 19.2%), using no comparator (3/26, 11.5%), no-intervention controls (2/26, 7.7%), other nontraditional teaching (2/26, 7.7%), and alternative digital tools (1/26, 3.8%). Overall, the predominantly positive findings in this field arise largely from study designs with limited methodological rigor and inconsistent use of validated measures, substantially reducing confidence in causal claims about technology effectiveness (Table 3).

**Table 3.** Comparator groups used in studies examining digital communication skills training in medical education that report beneficial effects of digital communication skills training.

Type of comparator	Total beneficial studies (n=81), n (%)^a^	Beneficial studies using validated measures (n=26), n (%)^b^
No comparator	20 (25)	3 (11.5)
Within-group	39 (48.8)	13 (50)
Traditional teaching	11 (13.8)	5 (19.2)
Alternative digital tool	3 (3.8)	1 (3.8)
Other nontraditional teaching	3 (3.8)	2 (7.7)
No-intervention control	4 (5)	2 (7.7)

a Percentages were calculated using the total number of studies reporting beneficial outcomes as the denominator (n=80).

b Percentages were calculated using the total number of beneficial studies using validated outcome measures as the denominator (n=26).

### Stage of Kolb’s Experiential Learning Cycle

Studies predominantly situated digital technologies within the early phases of Kolb’s experiential learning cycle [20] with 120 of 121 studies (99.2%) focusing solely on Stage 1 (Concrete Experience), Stage 2 (Reflective Observation), or both. Nearly one-half of studies (54/121, 44.6%) integrated both stages, typically through combinations of simulated encounters and structured feedback, making this the most common approach. Among studies focusing on a single stage, Stage 1 alone was addressed in 28.9% of studies (35/121), while Stage 2 alone was addressed in 25.6% (31/121).

Progression beyond these initial phases was virtually absent: No studies aligned primarily with Stage 3 (Abstract Conceptualization: 0/121, 0%), and only 1 study extended to Stage 4 (Active Experimentation: 1/121, 0.8%). Figure S8 in Multimedia Appendix 6 shows the distribution of studies across Kolb’s learning stages by technology type, demonstrating that all digital modalities concentrated overwhelmingly on Stages 1 and 2.

### Outcomes Mapped to Kirkpatrick’s Training Evaluation Model

All included studies reported outcomes that could be classified using Kirkpatrick’s 4-level model [21]. The vast majority focused on classroom or simulation-level outcomes: A significant number of studies (91/121, 75.2%) were evaluated at Level 2, measuring changes in learners’ knowledge, skills, or observed performance in simulated environments, while nearly all others (23/121, 19%) assessed Level 1 outcomes such as learner satisfaction, confidence, or perceived usefulness. Combined, 94.2% of studies (114/121) measured outcomes within educational settings rather than clinical practice.

In contrast, evaluation of real-world impact was minimal. Only 6 of the 121 studies (5%) assessed behavior change in clinical practice at Kirkpatrick’s Level 3, and 1 study (1/121, 0.8%) examined patient-level outcomes at Level 4. This concentration in Levels 1 and 2 indicates that, although there is substantial evidence for the educational effectiveness of digital technologies in controlled settings, the field has generated almost no evidence regarding their impact on clinical practice or patient outcomes. Figure S9 in Multimedia Appendix 7 presents the distribution of studies across technology types and Kirkpatrick levels, illustrating the concentration at Levels 1 and 2 with minimal progression to behavior change (Level 3) or patient outcomes (Level 4).

### AI in CST

A small but emerging subset of studies (15/121, 12.4%) [50,116,127,130,131,135-138,140,142-144,150,152] explicitly incorporated AI into CST, spanning undergraduate (11/15, 73.3%), postgraduate (3/15, 20%), and mixed learner populations (1/15, 6.7%). AI applications were concentrated predominantly within VP simulators (12/15, 80%) [116,127,130,131,135-138,140,142-144], with smaller numbers categorized under other digital interventions (2/15, 13.3%) [146,150] and video-recording approaches (1/15, 6.7%) [50]. These AI applications primarily simulated patient interactions and delivered automated feedback, supporting the development of skills such as history taking, information gathering, interview techniques, nonverbal behavior, and empathy.

Reported outcomes were largely positive, with 12 of the 15 studies (80%) demonstrating beneficial effects on communication skills, a higher success rate than digital technologies overall. However, the remaining studies showed more limited evidence: 1 reported mixed findings [131], 1 was inconclusive [116], and 1 other found outcomes equivalent to traditional actor-based training [142]. Notably, the study with mixed findings [131], which relied on self-reported outcomes, highlighted limitations in AI realism and did not provide clear evidence of impact, suggesting that the effectiveness of AI may depend on both implementation quality and measurement approach. The distribution of evidence across technology types, communication domains, learner levels, Kolb’s learning stages, and Kirkpatrick’s evaluation levels is presented in the evidence and gap map (Multimedia Appendix 7), which visually highlights areas of concentrated research and gaps in the current evidence base.

## Discussion

### Summary of Principal Findings

This review mapped the digital technologies used for CST and examined reported outcomes through the lens of experiential learning theory and translational evaluation frameworks. The evidence base has expanded substantially over the past decade, with notable acceleration following the COVID-19 pandemic, though research remains concentrated in North America and Europe and predominantly involves undergraduate learners. Three principal technology categories dominated the field: recording-based approaches, live-streaming platforms, and VP simulators. Application of Kolb’s experiential learning cycle [20] and Kirkpatrick’s evaluation model [21] revealed that current digital interventions overwhelmingly support early-stage learning processes and are evaluated almost exclusively within educational settings, with minimal evidence of progression to deeper competency development or translation into clinical practice and patient outcomes.

### Comparison of Findings and Interpretation Within the Context of Literature

The field of digital CST has undergone substantial transformation in recent years. Live-streaming platforms expanded markedly after 2020, likely accelerated by the shift to virtual learning environments during the COVID-19 pandemic, while VP simulators demonstrated the most striking recent growth. These trends mirror broader patterns observed in the adoption of digital tools in medical education [15] and in the expansion of virtual simulation for CST [17]. Despite this technological diversification, recording-based approaches that enable structured feedback and reflection continue to be widely used, demonstrating their continued relevance in CST.

Study content across technologies varied in ways that have implications for curriculum design. Recording-based tools and VP simulators were heavily focused on general communication and history taking, with more limited attention to areas such as shared decision-making, patient education, or communication with specific patient populations. Live stream platforms, by contrast, demonstrated greater diversity across communication domains, including telecommunication and remote consultation skills. This uneven exploration suggests that some modalities may be more readily adapted to diverse communication scenarios than others, though the reasons for these differences remain unclear and warrant further investigation.

The evidence base, although growing, reveals substantial methodological limitations that constrain confidence in reported outcomes. The vast majority of studies demonstrating beneficial effects relied on designs with limited capacity for causal inference, predominantly using within-group comparisons or lacking comparators entirely. Validation of outcome measures was similarly inconsistent, with most studies using nonvalidated tools. These patterns indicate that, although digital technologies show promise, the strength of evidence supporting their effectiveness remains weak [153]. Notwithstanding, live stream platforms showed both the highest rate of beneficial outcomes and greater diversity in the communication domains addressed, suggesting this modality may offer particular flexibility in supporting varied learning objectives [154,155]. Although most VP simulator studies demonstrated beneficial outcomes, a small number of studies reported outcomes equivalent to traditional methods, raising questions about when and for whom VPs offer advantages over established approaches. These patterns suggest that effectiveness may depend not only on the technology itself but also on how it is implemented, for what learning objectives, and with which learner populations [156].

Application of educational theory frameworks revealed two striking gaps in current research. First, digital technologies are almost exclusively used to support the initial phases of experiential learning (ie, concrete experience and reflective observation), with virtually no progression to abstract conceptualization or active experimentation. This concentration suggests that current technologies facilitate earlier learning processes for CST, stopping short of the deeper conceptual understanding and experimental application that characterize advanced skill development [20]. Second, outcome evaluation overwhelmingly focused on classroom and simulation-level measures, with minimal assessment of whether learning gains translate to actual clinical practice or patient outcomes. This near absence of real-world evaluation represents a critical knowledge gap of whether the educational benefits observed in controlled settings yield meaningful improvements in patient care [1,2].

The findings of this review are consistent with previous observations that research on CST in medical education is dominated by descriptive and quasi-experimental designs, with relatively few robust comparative studies [157]. Our analysis adds new depth by demonstrating that most studies reporting beneficial outcomes relied on within-group designs or lacked comparators entirely and validated outcome measures were used in few positive studies. This methodological profile reveals an evidence base that remains largely exploratory rather than definitive, a pattern that was particularly evident during the rapid post-COVID-19 adoption of digital tools, when educational continuity was prioritized over rigorous evaluation [15]. The heterogeneity in study designs and outcome measures continues to hamper synthesis of effectiveness claims across technologies [11].

AI-enabled CST represented a small proportion of the included studies, with most applications embedded within VP simulators and focused on automating patient interactions or feedback. Although many of these studies reported beneficial effects, few used validated communication outcome measures, and evidence was largely derived from noncomparative designs and proximal learning outcomes. Recent literature [158] has similarly noted that, although interest in AI-supported communication skills education is increasing, research to date has predominantly focused on feasibility and instructional potential, with limited attention to the psychometric validation of AI-specific assessment approaches. Together, these findings suggest that AI-supported CST remains at an early stage of evidence development, requiring more rigorous and theory-aligned evaluation to support meaningful comparison and wider implementation.

A recent meta-analysis of 3 randomized controlled trials [11] assessed digital technology to be as effective as traditional teaching for developing communication skills, though low-quality evidence limited confidence in the finding. Our review supported this conclusion but extends the evidence base by including a wider range of study designs and technologies and confirmed that many reports of benefit arise from weaker methodological approaches. The finding that a minority of studies included external comparators means that most claims of benefit rest on uncontrolled observations, providing weak grounds for judging whether digital approaches outperform, match, or fall short of established methods. Digital technologies therefore have potential in this field, but stronger study designs and robust outcomes, also advocated by Gilligan et al [6], are required to achieve meaningful conclusions and would enhance confidence for attributing gains directly to the interventions. Additionally, our study has illuminated the positioning of technologies for CST within educational pedagogy and translation into clinical outcomes. Evidence of enhancement was most apparent in formative learning contexts. Learners frequently reported increased confidence, satisfaction, and perceived competence, and studies using video recording or conferencing highlighted the value of repeated practice, self-reflection, and timely feedback. These features may offer advantages in certain learning domains, especially where opportunities for repeated practice, asynchronous reflection, or scalable feedback are limited in traditional classroom-based approaches, which typically emphasize role-play and face-to-face interactions [6,159].

Cook and Ellaway [19] previously proposed an evaluation framework to aid synthesis of technology-enhanced learning that focused on the implementation process. Alternatively, placing intervention assessment within education theory, as in this review, can aid comparison while additionally providing insight into the learning and teaching process, thus informing future curricula development. However, the concentration of outcomes at Kirkpatrick Levels 1 and 2 (satisfaction and skill demonstration in educational settings) [21] with minimal assessment of behavior change in practice or patient outcomes raises a fundamental question about translational impact. The persistent emphasis on proximal learning outcomes may reflect pragmatic constraints, as behavior change and patient-level outcomes are more difficult and costly to measure, but it leaves largely unanswered whether communication skills developed through digital training transfer to clinical practice and ultimately improve patient care. This gap between educational and clinical outcomes has been noted in wider educational research, which often prioritizes measures that are easier to capture [160]. However, the near-total absence of practice-level and patient-level evaluation in this field is particularly concerning given the fundamental premise that CST should ultimately enhance patient care. The application of Kirkpatrick’s framework in this review makes this gap explicit: Demonstrating that learners can perform skills in simulated settings does not establish that they will apply those skills effectively in real clinical encounters or that such application improves patient outcomes.

### Strengths and Limitations

To our knowledge, this is the first broad and comprehensive review of digital technologies for CST in both undergraduate and postgraduate medical education. Our systematic approach, guided by predefined eligibility criteria and mapped to established educational frameworks, enabled synthesis across diverse study designs and outcomes. This approach not only provided a structured view of where and how digital technologies are being applied within CST but also revealed patterns in how learning is being conceptualized and measured across different educational contexts.

Several limitations should be noted. This was a scoping review; therefore, a formal risk of bias or quality assessment was not undertaken. However, the extracted data clearly indicate that many studies were of low methodological quality, often relying on small samples, weak and noncomparative designs, and unvalidated outcome measures. The predominance of such designs limits the strength of causal claims that can be made about technology effectiveness. Across the included studies, no digital intervention was associated with a clear deterioration in communication skills. Where benefits were not observed, findings were most often neutral or mixed rather than negative. Although this may suggest that digital approaches are unlikely to cause harm, it may represent publication bias, outcome measurement limitations, and the methodological weaknesses evident across most included studies, which lack the rigor needed to detect potential adverse effects. In addition, rapid advances in technology mean that some emerging tools may not yet be captured in the published literature. Gray literature was not searched, and, although such sources often lack the detailed outcome reporting necessary for mapping to theoretical frameworks, this exclusion may have resulted in some relevant evidence being missed. However, the impact of this is likely mitigated by the large number of studies included in our review. Most included studies were conducted in North America or Europe, potentially limiting the generalizability of findings to developing countries, as infrastructure, personal cost, and institutional constraints shape how digital technologies are used in medical training globally [161].

Although Kolb’s experiential learning cycle provided a useful framework for examining which learning processes were targeted by digital CST interventions, we acknowledge that contemporary cognitive and neuroscience research suggests learning may occur nonlinearly, with stages overlapping or occurring simultaneously rather than sequentially [162-164]. In this review, all stages of Kolb’s cycle supported by each intervention were recorded, and we recorded multiple stages of learning when appropriate. More than one-half of the included studies supported both concrete experience and reflective observation, reflecting common pedagogical designs that combine simulated encounters with structured reflection. Despite allowing for overlap, very few studies extended to later stages of experiential learning, with almost no interventions supporting abstract conceptualization or active experimentation. This pattern, whether viewed through a sequential or nonlinear lens, indicates that digital technologies as currently designed and evaluated support initial skill acquisition and reflection but do not facilitate progression toward the conceptual understanding and experimental application needed for mastery.

### Conclusions and Recommendations

Digital CST interventions showed promise for supporting early-stage learner outcomes, such as improved confidence, satisfaction, and knowledge or skill acquisition. However, there was little evidence that these technologies supported progression to longer-term competency development in either undergraduate or postgraduate learners, and the translation of digitally acquired skills into improved patient communication or care remains largely unevaluated. Although these technologies offer scalable, flexible training opportunities, current evidence does not establish their effectiveness relative to traditional methods or their impact on clinical practice. Digital CST should be integrated thoughtfully within broader curricula and accompanied by rigorous evaluation extending beyond educational settings to assess impact on patient outcomes. Several priorities emerge for future research and practice. First, greater methodological rigor in evaluation is essential, including comparative designs that establish whether digital approaches match, exceed, or fall short of traditional training, and consistent use of validated outcome measures to ensure robust assessment. Second, research must extend beyond educational outcomes to assess whether learning gains translate to clinical practice and patient care to determine whether learning gains translate beyond educational settings. Third, investigation is needed into how digital technologies might support progression through complete learning cycles, moving beyond initial skill acquisition and reflection to support conceptual understanding and experimental application in varied contexts [162-164]. Finally, as AI-enabled approaches continue to emerge in CST, there is opportunity to design them not only as training tools but also as technologies that scaffold competency development and translation into improved patient outcomes.

## Supplementary material

10.2196/87012 Multimedia Appendix 1 Search strategy.

10.2196/87012 Multimedia Appendix 2 Blank data extraction form.

10.2196/87012 Multimedia Appendix 3 Summary table of data extracted from included studies by category of technology.

10.2196/87012 Multimedia Appendix 4 Distribution of studies examining digital communication skills training in medical education across learner groups by technology type and year of publication.

10.2196/87012 Multimedia Appendix 5 Validation status of assessment tools used in studies examining digital communication skills training in medical education, stratified by learner level.

10.2196/87012 Multimedia Appendix 6 Evidence gap map showing distribution of included studies across Kolb’s experiential learning cycle and Kirkpatrick’s evaluation model by technology type.

10.2196/87012 Multimedia Appendix 7 Evidence gap map of studies examining digital communication skills training in medical education.

10.2196/87012 Checklist 1 PRISMA-ScR (Preferred Reporting Items for Systematic Reviews and Meta-Analyses Extension for Scoping Reviews) checklist.

10.2196/87012 Checklist 2 PRISMA-S (Preferred Reporting Items for Systematic Reviews and Meta-Analyses Extension for Literature Searches) checklist.

## Abbreviations

AI: artificial intelligence
CINAHL: Cumulative Index to Nursing and Allied Health Literature
CST: communication skills training
ERIC: Educational Resource Information Center
JBI: Joanna Briggs Institute
PRISMA-S: Preferred Reporting Items for Systematic Reviews and Meta-Analyses Extension for Literature Searches
PRISMA-ScR: Preferred Reporting Items for Systematic Reviews and Meta-Analyses Extension for Scoping Reviews
VP: virtual patient
