# Supplementary material for: Use of Digital Technology for Developing Communication Skills in Undergraduate and Postgraduate Medical Education: Scoping Review
Source: JMIR Med Educ. 2026 Apr 20;12:e87012. doi: 10.2196/87012 (PMC13094807; doi:10.2196/87012)
Supplement: Multimedia Appendix 1 [file mededu-v12-e87012-s001.docx]

**Appendix S1. Full electronic search strategies presented exactly as run.**

**Medline**

Initial search: November 2024
Updates: August 2025; 5 January 2026

1. exp education, professional/ not education, veterinary/
2. exp education, medical/
3. medical training.mp
4. ((medic* or premed* or pre-med* or pre-regist* or preregist* or student* or undergraduate* or intern or post-regist* or postregist* or graduate* or postgraduate* or post-graduate* or residen* or clinician* or doctor* or physician*) ADJ (education or degree* or course* or program* or curricul* or learn* or teach* or instructor* or instruction)).mp.
5. 1 or 2 or 3 or 4
6. Computer-Assisted Instruction/
7. exp Computer Simulation/
8. exp Simulation Training/
9. ((patient* or clinical or medical) adj2 (simulat* or model*)).mp.
10. “virtual patient”.mp.
11. (computer* or digital* or tech or technolog* or distance or remote* or electronic or mobile or online* or interactiv* or multimedia or multi-media or internet or web* or virtual* or “VR” or “augmented reality” or “artificial intelligence” or “mixed reality” or game* or gaming or videogram*).mp.
12. (simulat* adj3 (course* or educat* or instruct* or learn* or train* or teach* or platform* or high-fidelity)).mp.
13. ((educat* or instruct* or learn* or simulat* or train* or teach* or interactiv*) adj2 (technolog* or tech)).mp.
14. 6 or 7 or 8 or 9 or 10 or 11 or 12 or 13
15. 5 and 14
16. (communicat* adj4 (skill* or ability* or competen* or train* or educat* or curricul* or material or learn* or teach* or program* or course)).mp.
17. 15 and 16

**Embase via OVID.**

Initial search: November 2024
Updates: August 2025; 5 January 2026

1. exp education, professional/ not education, veterinary/
2. exp education, medical/
3. medical training.mp
4. ((medic* or premed* or pre-med* or pre-regist* or preregist* or student* or undergraduate* or intern or post-regist* or postregist* or graduate* or postgraduate* or post-graduate* or residen* or clinician* or doctor* or physician*) ADJ (education or degree* or course* or program* or curricul* or learn* or teach* or instructor* or instruction)).mp.
5. 1 or 2 or 3 or 4
6. Computer-Assisted Instruction/
7. exp Computer Simulation/
8. exp Simulation Training/
9. ((patient* or clinical or medical) adj2 (simulat* or model*)).mp.
10. “virtual patient”.mp.
11. (computer* or digital* or tech or technolog* or distance or remote* or electronic or mobile or online* or interactiv* or multimedia or multi-media or internet or web* or virtual* or “VR” or “augmented reality” or “artificial intelligence” or “mixed reality” or game* or gaming or videogram*).mp.
12. (simulat* adj3 (course* or educat* or instruct* or learn* or train* or teach* or platform* or high-fidelity)).mp.
13. ((educat* or instruct* or learn* or simulat* or train* or teach* or interactiv*) adj2 (technolog* or tech)).mp.
14. 6 or 7 or 8 or 9 or 10 or 11 or 12 or 13
15. 5 and 14
16. (communicat* adj4 (skill* or ability* or competen* or train* or educat* or curricul* or material or learn* or teach* or program* or course)).mp.
17. 15 and 16

**Cinahl via Ebsco Host.**

Initial search: November 2024
Updates: August 2025; 5 January 2026

1. Medical student or physician or medical doctor

2. Medical education

3. 1 and 2

4. Virtual patient or (computer simulations or simulation based learning) or case based learning or (problem based learning or pl or problem-based-learning) or (clinical simulation or simulation training or patient simulation)

5. 3 and 4

6. communication skills

7. 5 and 6

**ERIC via Ebsco Host.**

Initial search: November 2024
Updates: August 2025; 5 January 2026

1. Medical student or physician or medical doctor

2. Medical education

3. 1 and 2

4. Virtual patient or (computer simulations or simulation based learning) or case based learning or (problem based learning or pl or problem-based-learning) or (clinical simulation or simulation training or patient simulation)

5. 3 and 4

6. communication skills

7. 5 and 6
