# Supplementary material for: Use of Digital Technology for Developing Communication Skills in Undergraduate and Postgraduate Medical Education: Scoping Review
Source: JMIR Med Educ. 2026 Apr 20;12:e87012. doi: 10.2196/87012 (PMC13094807; doi:10.2196/87012)
Supplement: Multimedia Appendix 3 [file mededu-v12-e87012-s003.docx]

Appendix S5. Summary table of data extracted from included studies* by category of technology

| Category of digital technology and author, year of publication | Educational Context | Aim of Study | Digital Technology and its application | Outcome of Digital Technology use |
| --- | --- | --- | --- | --- |
| Recording-based approaches (n=51) | | | | |
| Cals et al, 2007 [34] | Postgraduate; General Practitioners (GPs) | To determine if GPs trained in the enhanced communication skills could apply these skills in a routine practice setting, and whether GPs could maintain these acquired skills over the longer-term. | Audio Recording; Used to record simulated telephone encounters for individual or group feedback sessions during clinical duties | Beneficial |
| Evens et al,  1983 [39] | Postgraduate; First to third year Resident Doctors | To describe an audiotaped simulation program | Audio Recording; Used to record student consultations for later self-reflection and discussion during a communication course | Inconclusive |
| Roter et al, 1995 [66] | Postgraduate; Primary Care Physicians | To explore the effect of communication skills training on the process and outcome of care associated with patient' emotional distress | Audio Recording; Used to record consultations between students and standardized patients (SPs) using a language translation app across language barriers | Beneficial |
| Beyth et al, 2009 [30] | Postgraduate; Gynaecologists | To train gynaecologists in communication with adolescent patients using simulated consultations | Video Recording; Used to capture simulated consultations for debrief and assessment | Inconclusive |
| Bonnaud-Antignac et al, 2010 [31] | Undergraduate; Fifth year medical students | To evaluate a course on cancer diagnosis disclosure for fifth-year medical students. | Video Recording; Used to record simulated consultations for evaluation of protocol adherence | Mixed |
| Bos-vanden Hoek et al, 2023 [32] | Postgraduate; Medical oncologists in training | To compare the effectiveness of blended online training with face-to-face training for oncologists on shared decision making in palliative care | Video Recording; Used to assess simulated patient interactions before and after online training | Beneficial |
| Busenius et al, 2022 [33] | Undergraduate; fifth- and sixth-year medical students | To test the reliability of the Global Rating Scale for assessing communication in video-recorded encounters | Video Recording; Used to capture simulated consultations for rating communication skills | Inconclusive |
| Carrard et al, 2020 [35] | Undergraduate; Fourth year medical students | To examine the impact of individualized breaking bad news training on observer-rated performance | Video Recording; Used to record student-SP interviews for feedback and reflection | Beneficial |
| Denizon Arranz et al, 2021 [36] | Undergraduate; Third year medical students | To describe an experiential training method using standardized patient interviews | Video Recording; Used to capture student interviews with SPs for annotation, peer, and self-feedback | Beneficial |
| Dohms et al, 2020 [37] | Postgraduate; First year medical residents in primary care | To explore perceptions and outcomes of video-based feedback in formative assessment | Video Recording; Used to record resident consultations, later reviewed with peers and facilitators for reflection and feedback | No effect |
| Dowling et al, 2007 [38] | Postgraduate; Specialty registrars | To evaluate feasibility of video-based formative assessment tool | Video Recording; Used to record trainee consultations, later assessed by clinical trainers and questionnaires | Beneficial |
| Farnill et al, 1997 [40] | Undergraduate; Second year medical student | To develop interviewing and multicultural communication skills using video review | Video Recording; Used to capture student interviews for group observation, discussion, and reflection | No effect |
| Fischbeck et al,  2020 [41] | Undergraduate; Second semester medical students | To evaluate effectiveness of digitized asynchronous physician–patient communication course | Video Recording; Used to capture roleplay simulations, later reviewed with task-based checklists for feedback | Beneficial |
| Freytag et al,  2022 [42] | Postgraduate; Physicians, fellows, residents, nurse practitioners | To assess feasibility of an interpersonal communication skills training program | Video Conferencing; Used to stream and record consultations for feedback using video clips | Inconclusive |
| Hardoff et al, 2016 [43] | Postgraduate; Paediatricians and family physicians | To describe simulation-based program to improve communication with adolescents and parents | Video Recording; Used to capture actor-patient encounters for analysis and debriefing | Beneficial |
| Harendza et al, 2023 [45] | Undergraduate; Final year medical students | To establish training integrating communication and clinical reasoning skills | Video Conferencing; Used to record simulated patient consultations with feedback from SPs and faculty | Beneficial |
| Harnof et al, 2013 [44] | Postgraduate; Neurosurgery residents | To assess national simulation-based communication training for neurosurgeons | Video Recording; Used to capture role-play consultations for feedback by neurosurgeon and communication expert | Beneficial |
| Hulsman et al, 2009 [46] | Undergraduate; Year 2 medical students | To assess reflection and perceived learning in communication | Video Recording; Used to record history-taking consultations for peer review, reflection, and group feedback | Mixed |
| Hulsman et al, 2015 [47] | Undergraduate; Year 4 medical students | To explore characteristics of self-evaluations and peer feedback on communication skills | Video Recording; Used to capture student-SP consultations for annotation, peer review, and self-assessment | Mixed |
| Kaltman et al, 2018 [48] | Undergraduate; First year medical students, communication class | To evaluate immersive video simulation for basic communication skills training | Video Recording; Used to deliver interactive video cases with branching responses and feedback | Beneficial |
| Knowles et al, 2001 [49] | Undergraduate; Fourth year students in genitourinary attachment | To determine effectiveness of roleplay with feedback in improving communication | Video Recording; Used to record history-taking exercises for self, peer, and tutor feedback | Beneficial |
| Kobayashi et al, 2023 [50] | Postgraduate; Physicians caring for older adults | To analyse communication skills of physicians using AI and explore training benefits | Video Recording; Used to capture simulated patient encounters with eye-tracking and fixed cameras, analysed with AI | Beneficial |
| Liu et al, 2016 [51] | Undergraduate; Second year medical students, clinical communication skills course | To evaluate EQ-Clinic in improving clinical communication skills | Video Recording; Used to record teleconsultations with SPs, with feedback using automated analysis and human review | Beneficial |
| Makaricheva et al, 2024 [52] | Undergraduate; Fifth- and sixth-year medical students | To assess effectiveness of communication skills training | Video recordings of student consultations with actor-simulated patients. Roles reversed and videotaped; students later watched their own videos. | Beneficial |
| Mauksch et al, 2013 [53] | Undergraduate; Third, fourth- or fifth-year medical students | To test methods that extend communication training into the fourth year | Video recordings of student consultations with real patients used for self-reflection in communication training. | Beneficial |
| Mohos et al, 2020 [54] | Undergraduate; Fourth year medical students | To evaluate online teaching of communication skills | Video-conferenced consultations with simulated patients via Zoom, recorded and reviewed in group discussions. | Inconclusive |
| Moulton et al, 2009 [55] | Mixed; Fourth year students and junior residents | To assess effectiveness of Integrated Procedural Performance Instrument (IPPI) format as a teaching tool for communication | Videotaped simulated patient scenarios (wound closure, catheterisation); feedback from SPs via video and ratings by independent assessors. | Beneficial |
| Muller et al, 2019 [56] | Postgraduate; Physicians in asthma care | To evaluate impact of SDM training on physician behaviours and perceptions | Videotaped consultations with asthma patients; reviewed in small groups with video analysis, feedback, discussions; followed by second videotaped consultation and telephone coaching. | Beneficial |
| Naik et al, 2018 [57] | Undergraduate; Fourth year medical students | To describe a simulation-based telemedicine curriculum | Videotaped student consultations with standardised patients during telemedicine curriculum; assessed with checklist and tutor debrief. | Inconclusive |
| Noordman et al, 2011 [58] | Postgraduate; Experienced primary care physicians | To describe video-feedback method for physician communication skills | Videotaped physician consultations; later reviewed with written reports and feedback by phone/in person based on patient opinions and communication scores. | Inconclusive |
| Noordman et al, 2019 [59] | Postgraduate; Residents in mixed adult specialities | To evaluate a patient-centred communication and empathy training course | Presentations and videotaped difficult consultations with actors, followed by reflection. Residents repeated videotaped consultations with real patients at 2 months. | No effect |
| Ohlmann et al, 2024 [60] | Undergraduate; First year medical students in psychology module | To describe a module teaching placebo and nocebo effects with communication | Video Recording; Used to video role-play conversations between doctor, patient, and family for group viewing, discussion, and feedback | Beneficial |
| Ozcakar et al, 2009 [61] | Undergraduate; Second year medical students in clinical skills program | To test whether video feedback improves history-taking and communication skills | Video Recording; Used to record simulated patient interviews with checklist assessment, student self-review, and verbal feedback before repeat consultation | Beneficial |
| Perron et al, 2015 [62] | Undergraduate; Second- and third-year medical students | To evaluate differences between direct and video-based feedback formats | Video Recording; Used to compare immediate tutor feedback on live consultations with feedback after reviewing recorded consultations | Beneficial |
| Pless et al,  2021 [63] | Undergraduate; Fourth-year medical students | To evaluate self and peer annotations with feedback in communication course | Video Recording; Used to record student–simulated patient interviews with peer annotations, followed by group discussion of annotated clips | Inconclusive |
| Ravitz et al, 2013 [64] | Postgraduate; Family medicine trainees (1–3 years postgrad) | To test interactive medical education using coaching and standardized patients | Video Recording; Used to videotape standardized patient interviews with subsequent individualized coaching from psychiatrists | Beneficial |
| Roter et al, 2004 [65] | Postgraduate; First-year paediatric residents | To explore acceptability of innovative video feedback for communication training | Video Recording; Used to record resident–simulated patient consultations, with feedback via didactic teaching, role play, interactive CD-ROM, and repeat assessment | Beneficial |
| Ruesseler et al, 2017 [67] | Undergraduate; Fourth-year medical students | To compare video and oral feedback in surgical communication training | Video Recording; Used to video student role-plays with simulated patients, followed by feedback using the recording or orally | Beneficial |
| Scardovi et al,  2003 [68] | Postgraduate; Established GPs | To evaluate efficacy of training on GP interview skills and distress detection | Video Recording; Used to record GP consultations for discussion and feedback during structured teaching sessions | Beneficial |
| Setubal et al, 2018 [69] | Postgraduate; Perinatology residents (1st to 4th year) | To assess whether structured sessions enhance communication in breaking bad news | Video Recording; Used to record SP consultations for immediate feedback, followed by randomisation to video review or no further training | No effect |
| Slort et al, 2014 [70] | Postgraduate; Third-year general practice trainees in palliative care | To evaluate effectiveness of Availability, Current issues, and Anticipation (ACA) training to improve communication skills | Video Recording; Used to record simulated consultations with feedback, personal learning goals, and role-play practice | No effect |
| Smith et al, 2002 [71] | Postgraduate; Neurology registrars | To evaluate usefulness of videotaped consultations with simulated patients | Video Recording; Used to video SP consultations in neurology for review with facilitators, consultants, peers, and simulated patients | Beneficial |
| Smith et al, 2023 [72] | Postgraduate; Critical care and internal medicine subspecialty fellows | To assess impact of Simulation-Based Mastery Learning (SBML) training on breaking bad news skills | Video Recording; Used to record serious news delivery scenarios with SPs, followed by structured feedback and SP-based workshops | Beneficial |
| Supiot et al, 2008 [73] | Undergraduate; Fifth year medical students | To evaluate pilot communication course for breaking bad news | Video Recording; Used to record simulated interviews assessed via checklist, followed by individualised feedback from a psychologist and physician | Beneficial |
| Trent et al, 2015 [74] | Postgraduate; Paediatric trainees | To evaluate feasibility of adolescent-focused communication simulation | Video Recording; Used to video SP consultations with feedback from faculty and SP, followed by video review | Mixed |
| Van Rossem et al, 2019 [75] | Undergraduate; Family physician students in final year | To describe characteristics of training game | Video Recording: Used to video simulated consultations and home visits in a training game, followed by peer and faculty feedback | Beneficial |
| White et al, 2024 [76] | Postgraduate; Second year internal and family medicine residents | To evaluate effectiveness of video-based communication assessment (VCA) | Video Recording; Used to record VCA cases and provide delayed feedback via app after 2 weeks; control group received no feedback | Beneficial |
| Xiao et al,  2025 [80] | Postgraduate; Radiology Residents | To evaluate the efficacy of a smart glass (SG)-based communication skills training curriculum for radiology  residents in China | Video Recording; Standardized patients wore smart glasses to record first-person video of resident–patient interactions. These recordings were then reviewed during structured debriefing sessions, where residents received targeted feedback. | Beneficial |
| Yuan et al, 2019 [77] | Postgraduate; Paediatric residents | To assess residents’ skills in breaking bad news pre- and post-simulation | Video Recording; Used to video SP simulations of BBN before and after training, with assessments using a validated scale | Beneficial |
| Zick et al, 2007 [78] | Undergraduate; First year medical students | To examine content of student self-assessments of communication skills | Video Recording; Used to record weekly patient–student interactions with structured feedback and self-review of videos after a final simulated patient encounter | Mixed |
| Levine et al,  2025 [79] | Postgraduate; Oncology residents | To assess feasibility of a randomized controlled trial (RCT) comparing different training experiences. | Video Recording; Used to record simulated serious illness conversations with standardized patients before and after an AI-supported communication training programme, with recordings reviewed to assess feasibility, learner performance, and perceived usefulness of structured feedback | Beneficial |
| Live-streaming platforms (n=33) | | | |  |
| Abraham et al,  2021 [81] | Undergraduate; Second year medical students, problem-based learning | To assess whether remotely facilitated face-to-face training is effective | Video Conferencing; Used for online simulated consultations via Zoom with peer observation and facilitator feedback | Mixed |
| Afonso et al,  2020 [82] | Undergraduate; First year medical students, respiratory module | To design an online interactive case-based respiratory module | Video Conferencing; Used for an online respiratory module combining demonstration, telemedicine encounter, and discussion | Beneficial |
| Aluce et al,  2024 [83] | Postgraduate; First-year residents in Emergency Medicine/Internal Medicine | To address scalability barriers in SBML | Video Conferencing; Used for online disclosure practice, workshops, and roleplay sessions with feedback | Beneficial |
| Bittner et al,  2016 [84] | Undergraduate; Fourth year or higher medical students | To assess whether translating documents into plain language improves communication | Video Conferencing; Used for online consultations with simulated patients via Skype and group assessment | Beneficial |
| Booth et al, 2022 [85] | Undergraduate; Final year medical students, obstetrics & gynaecology placement | To evaluate impact and transferability of telemedicine skills training | Video Conferencing; Used for Teams-based telemedicine consultations streamed to peers and facilitator with feedback | Beneficial |
| Bramstedts et al,  2014 [86] | Undergraduate; Second year medical students, nephrology module | To assess feasibility of ethics teaching via telemedicine | Video Conferencing; Used for student-SP interviews via Skype with peer observation | Beneficial |
| Clever et al, 2003 [87] | Postgraduate; Orthopaedic surgery trainees | To test if SPs could assess surgeons’ informed decision-making skills via video | Video Conferencing; Used for one-on-one SP consultations assessed with IDM checklist and feedback | Beneficial |
| Daetwyler et al, 2010 [88] | Undergraduate; Medical interns registered on an online module | To assess the effectiveness of an online communication skills module with added standardized patient feedback via video conference. | Video Conferencing; Used for SP encounters via videoconference with checklist and feedback | Beneficial |
| Deming et al, 2024 [89] | Postgraduate; Family medicine residents | To assess if telehealth workshops improve communication | Video Conferencing; Used for online workshops with SP roleplays and discussions of challenging communication tasks | Beneficial |
| Godoy-Pozo et al, 2023 [90] | Undergraduate; Third-year medical students | To enhance competencies using remote simulation | Video Conferencing; Used for synchronous SP simulations with clinical interview and structured debrief | Beneficial |
| Gur et al, 2024 [91] | Undergraduate; ENT module students | To compare self-assessment of communication after teaching | Video Conferencing; Used for online modules with video consultations, feedback, and group reflection | Beneficial |
| Hayes et al,  2025 [92] | Undergraduate; Final year medical students | To analyse student perceptions of an online course for communication skills | Video Conferencing; Used for online roleplay, peer feedback, and structured clinical assessments with SPs | Mixed |
| Heller et al,  2023 [93] | Postgraduate; Internal medicine residents | To provide training on telemedicine communication with patient feedback | Video Conferencing; Used for standardized patient post-discharge assessments with checklists and feedback | Mixed |
| Holmes et al,  2020 [94] | Postgraduate; Nephrology fellows | To assess impact of virtual workshops on communication skills | Video Conferencing; Used for online SP workshops with didactic sessions and simulated patient interviews | Mixed |
| Iammeechai et al,  2025 [95] | Undergraduate; Fourth year medical students, psychiatry module | To assess confidence in communication after online discussions and roleplay | Video Conferencing; Used for videoconference sessions with roleplay and discussion of sensitive communication scenarios | Beneficial |
| Jones et al,  2025 [96] | Postgraduate; Internal medicine residents (years 1–3) | To describe a telemedicine curriculum | Video Conferencing; Used for teaching sessions and observation of patient encounters with immediate feedback | Mixed |
| Khawand-Azoulay et al,  2025 [97] | Undergraduate; Fourth year, transitioning to residency course | To evaluate curriculum on family meetings at end of life | Video Conferencing; Used for online roleplay of family meetings with facilitator debrief | Beneficial |
| Knie et al,  2020 [98] | Undergraduate; Second year medical students | To develop and evaluate digital training for communication during COVID-19 | Video Conferencing; Used for asynchronous learning and synchronous roleplays with simulated patients via Zoom | Beneficial |
| Lenes et al,  2020 [99] | Undergraduate; History taking, psychiatry, and clinical competence courses | To assess feasibility of moving SP teaching online | Video Conferencing; Used for online simulated patient consultations with peer observation and feedback | Inconclusive |
| Mack et al,  2025 [100] | Postgraduate; Paediatric haematology and oncology fellows | To assess feasibility and learner value of a formal communication course | Video Conferencing; Used for small-group skills practice with standardized patients and feedback | Beneficial |
| Newcomb et al,  2021 [101] | Undergraduate; Fourth year surgical module students | To identify techniques to build relationships during video consults | Video Conferencing; Used for roleplay consultations with simulated patients and group debrief via Zoom | Beneficial |
| Newcomb et al,  2022 [102] | Undergraduate; Years 1–2 medical students | To describe curriculum teaching virtual communication skills | Video Conferencing; Used for teaching sessions with roleplay, recorded consultations, and reflection | Beneficial |
| Pang et al,  2021 [103] | Undergraduate; Third year medical students, surgery module | To evaluate online module for teaching informed consent | Video Conferencing; Used for small-group videoconference sessions with standardized patients and feedback | Beneficial |
| Phillips et al,  2023 [104] | Postgraduate; Primary care and internal medicine residents (years 1–3) | To evaluate standardized patient video consultations for performance feedback | Video Conferencing; Used for announced standardized patient consultations with immediate structured feedback | Beneficial |
| Pozo et al, 2025 [105] | Undergraduate; Second year medical students | To teach telehealth communication skills and assess satisfaction | Video Conferencing; Used for Zoom-based telehealth consultations with facilitator and peer debrief | Beneficial |
| Rasalam et al,  2020 [106] | Undergraduate; Fifth year medical students | To demonstrate virtual clinics as authentic clinical learning | Video Conferencing; Used for simulated patient consultations over Zoom with tutor observation and feedback | Beneficial |
| Rivet et al,  2023 [107] | Mixed; Final year medical students, emergency medicine interns, general surgery interns | To train residents in virtual communication and assess performance | Video Conferencing; Used for videoconference simulations with standardized patients and feedback from SPs and facilitator | No effect |
| Ruddock et al, 2021 [108] | Undergraduate; Psychiatry students | To evaluate an immersive simulation course for psychiatry training | Video Conferencing; Used for live video broadcasts of simulated clinical scenarios with peer and facilitator debrief | Beneficial |
| Sasnal et al,  2021 [109] | Postgraduate; Neurology residents | To test feasibility of virtually coaching residents on communication | Video Conferencing; Used for real-time patient consultations with tutor observation and debrief | Beneficial |
| Taylor et al,  2024 [110] | Postgraduate; Rural and remote doctors | To evaluate a telehealth training program (TCSP) for rural doctors | Video Conferencing; Used for scenario-based remote consultation training with interactive video sessions and feedback | Beneficial |
| Tsui et al,  2024 [111] | Undergraduate; Fifth year medical students, psychiatry module | To evaluate effectiveness of online small-group interview training | Video Conferencing; Used for simulated telehealth consultations with SPs across remote video links, followed by feedback and assessment | Mixed |
| Yudkowsky et al, 2011 [112] | Postgraduate; First-year surgery residents | To pilot a simulated telehealth encounter to explore communication skills | Video Conferencing; Used for clinical interview workshops with standardized patients and peer feedback via teleconference | Mixed |
| Geng et al,  2025 [113] | Postgraduate; Second year clinical postgraduates | To compare the efficacy of online versus offline CST in postgraduate medical students. | Video Conferencing; Used to deliver an online CST with role-play, small-group practice, and Balint group discussions conducted via videoconferencing software with mandatory camera use, enabling interactive communication skills practice and feedback | Beneficial |
| Virtual Patient Simulators (n=32) | | | |  |
| Andrade et al, 2010 [114] | Postgraduate; Geriatric medicine and internal medicine specialty trainees | To test feasibility of creating SP avatars for training in breaking bad news | Virtual Patient Simulators; Used for avatar-based encounters with standardized patients recorded via screen capture | Beneficial |
| Bearman et al, 2001 [115] | Undergraduate; Pre-clinical students | To compare student attitudes toward different VP designs | Virtual Patient Simulators; Used for presentation of two VP case models for comparison | Inconclusive |
| Borg et al,  2024 [116] | Undergraduate; Third year medical students in rheumatology placements | To explore added value of LLM-enhanced robotic VP vs conventional VP | Virtual Patient Simulators; Used for VP cases on a social robotic platform with LLM support | Inconclusive |
| Bruen et al, 2017 [117] | Undergraduate; Psychiatry module students | To explore feasibility of adaptive simulations for learning and assessment | Virtual Patient Simulators; Used adaptive platform (SkillSims) for psychiatric consultations with decision points and analytics-based feedback | No effect |
| Carrard et al, 2020 [118] | Undergraduate; Fourth year medical students | To explore value of VP simulation in breaking bad news training | Virtual Patient Simulators; Used for virtual consultation rooms with VP avatars, allowing repeated practice and video review | Inconclusive |
| Courteille et al, 2014 [119] | Undergraduate; Sixth semester medical students (clinical level) | To investigate interpersonal dynamics in VP encounters | Virtual Patient Simulators; Used interactive free-text patient history-taking with video clips of SPs simulating different conditions | Beneficial |
| Detering et al, 2014 [120] | Postgraduate; General practitioners and doctors-in-training | To develop and evaluate interactive ACP programme | Virtual Patient Simulators; Used simulated ACP scenarios with e-simulation, DVDs, and workshops incorporating feedback and roleplay | Beneficial |
| Dickerson et al,  2006 [121] | Undergraduate; Second- and third-year medical students | To test appropriateness of synthesized speech for VPs | Virtual Patient Simulators; Used avatar patient with scripted history and recorded speech | Equivalent |
| Foster et al,  2015 [122] | Undergraduate; Second year medical students | To evaluate VP simulation for teaching suicide risk assessment | Virtual Patient Simulators; Used text-based VP simulation of mental health scenarios, compared with video learning | Mixed |
| Frey-Vogel et al,  2022 [123] | Postgraduate; Pediatric residents | To assess acceptability and realism of avatar patients | Virtual Patient Simulators; Used live-actor-controlled animated avatars via Zoom for communication practice | Inconclusive |
| Jacklin et al, 2021 [124] | Undergraduate; Medical students at a conference | To evaluate VP workshop for shared decision-making | Virtual Patient Simulators; Used onscreen avatar with multiple-choice interactions and personalized feedback | Beneficial |
| Kleinsmith et al,  2015 [125] | Undergraduate; Third year medical students | To test if students respond empathetically to VP concerns | Virtual Patient Simulators; Used interactive video Simulators with branching choices and feedback | Beneficial |
| Kron et al,  2017 [126] | Undergraduate; Second year medical students | To assess communication skills using VP scenarios vs multimedia module | Virtual Patient Simulators; Used virtual human scenarios with individualized feedback on choices and nonverbal behaviours | Beneficial |
| McCarrick et al, 2025 [127] | Undergraduate; Final year medical students, surgical module | To evaluate usefulness of structured VP interactions alongside immersion learning | Virtual Patient Simulators; Used ChatGPT-based simulated histories combined with standard teaching | Beneficial |
| McCarthy et al,  2022 [128] | Postgraduate; Emergency medicine residents (years 1–4) | To evaluate app for teaching diagnostic uncertainty communication | Virtual Patient Simulators; Used serious game app with actor-based simulations and patient response choices | No effect |
| Mool et al,  2024 [129] | Undergraduate; Second year problem-based learning module | To explore student interactions with generative AI simulated patients | Virtual Patient Simulators; Used AI-enabled avatars for history-taking in 3D exam room with voice-to-voice interaction and feedback | Beneficial |
| Mukadam et al,  2025 [130] | Undergraduate; Fourth year clinical placement | To examine perceptions of ChatGPT as simulated patient | Virtual Patient Simulators; Used ChatGPT roleplay scenarios with structured feedback on communication skills | Beneficial |
| Poulose et al,  2025 [131] | Mixed; Medical students and recent graduates (FY1) | To evaluate AI-simulated patients vs traditional peer-to-peer formats | Virtual Patient Simulators; Used SIMPAT system generating AI-simulated patient scenarios with programmed responses | Mixed |
| Raafat et al,  2024 [132] | Undergraduate; Fourth year medical students | To test if Klark tool improves student confidence and competence | Virtual Patient Simulators; Used interactive virtual cases with SP consultations pre- and post-training | Beneficial |
| Sezer et al,  2019 [133] | Undergraduate; Not stated | To design and evaluate a 3D VP application for communication | Virtual Patient Simulators; Used avatar-based consultation program with assessments | Equivalent |
| Thompson et al, 2025 [134] | Mixed; Medical students and residents | To test VR training module on implicit bias in communication | Virtual Patient Simulators; Used VR headset with conversational AI patient for practice scenarios | Beneficial |
| Wang et al,  2025 [135] | Undergraduate; Fifth year medical students | To assess effectiveness of GPT-based simulated patients for history-taking | Virtual Patient Simulators; Used GPT-4 powered AI patients for repeated clinical scenarios with text-based feedback | Beneficial |
| Yamamoto et al,  2024 [136] | Undergraduate; Fourth year medical students | To test whether AI-simulated patients improve interview skills | Virtual Patient Simulators; Used GPT-4 powered chat-based patient platform for interview practice with automated feedback | Beneficial |
| Chiu et al,  2025 [137] | Undergraduate; Second year medical students in osteopathic medicine | To evaluate the impact of engaging ChatGPT in a predesigned primary care case featuring a BBN scenario on medical students’ perceived learning to deliver BBN | AI Chatbot simulator; Used for written role-play breaking-bad-news scenarios where students interacted with an AI-simulated patient and received immediate rubric-based feedback on communication skills | Beneficial |
| Comulada et al,  2025 [138] | Undergraduate; Medical students | To determine whether the VP-enabled simulation could provide a  realistic, emotionally engaging, and technically reliable learning experience that complements existing SP training. | Virtual Patient Simulator: Used to deliver VP-enabled communication simulations designed to provide a realistic, emotionally engaging, and technically reliable learning experience to complement existing standardized patient training | Beneficial |
| Davidovics et al,  2025 [139] | Undergraduate; Medical students | To explore the impact of VR Patients™-Meta Quest 3 simulations in clinical communication and decision-making among medical students | Virtual Patient Simulator: Used to provide VP-based communication skills training with interactive simulated clinical encounters, evaluated for learner engagement, realism, and educational value, alongside facilitated discussion and feedback | Beneficial |
| Herschbach et al,  2025 [140] | Postgraduate; Physicians | To investigate the capability of an AI-based chatbot providing physicians with real-time feedback to train communication techniques. | AI Chatbot: Used to deliver real-time, automated feedback on physicians’ communication techniques during simulated text-based clinical conversations, with evaluation of feedback accuracy, perceived usefulness, and short-term behavioural change | Beneficial |
| Jadoon et al,  2025 [141] | Mixed; Undergraduate MBBS trainees and Postgraduate FCPS/MD trainees | To evaluate the effectiveness of virtual patient-based simulation in enhancing key communication skills and to explore learner  experience. | Virtual Patient Simulator; Used to deliver interactive, case-based virtual patient simulations for CST, with learners completing repeated simulated encounters and pre–post self-reported assessments of communication skills, confidence, and learner experience. | Beneficial |
| Lee et al,  2025 [142] | Undergraduate; Fourth year medical students in clinical years | To share the practical experiences and lessons learned from conducting an RCT with a novel AI educational tool, and second, to conduct a preliminary comparison of the educational effects of AI chatbot-based simulation and traditional actor-based teaching. | AI Chatbot Simulator; Used to deliver AI-simulated patient conversations for communication skills training, with learner performance and perceptions compared against traditional simulation methods | Equivalent |
| Suarez-Garcia et al,  2025 [143] | Undergraduate; Medical students | To evaluate whether a VR based simulation could improve students’ ability to disclose a type 2 diabetes mellitus (T2DM) diagnosis with clarity, structure, and empathy. | AI Virtual Patient (Generative AI); Used for asynchronous diagnostic and communication skills training through repeated interactions with a generative AI virtual patient, providing automated, personalised feedback with pre–post performance assessment. | Beneficial |
| Tyrell et al,  2025 [144] | Undergraduate; Third year medical students | To compare a fully automated AI-driven voice recognition-based virtual patient simulator with traditional actor-based consultation skills simulated training for differences in developing self-rated communication skills, student satisfaction scores, and direct cost comparison. | AI-Driven Virtual Patient Simulator; Used to deliver asynchronous, web-based spoken consultation simulations with unrestricted two-way dialogue and automated feedback, compared with actor-based simulation in a randomised crossover design. | Beneficial |
| Young et al,  2025 [145] | Undergraduate; Fourth year medical students | To assess the impact of a VR curriculum on attitudes and  confidence around motivational interviewing (MI) competencies and measure related implementation outcomes. | Virtual Patient Simulator; Used to deliver remote immersive simulated patient encounters via VR to support medical students’ communication skills practice, followed by structured group debriefing and feedback | Beneficial |
| Other (n=5) | | | | |
| Ba et al,  2024 [146] | Undergraduate; Pediatric medical internship (2 weeks) | To evaluate effectiveness of ChatGPT-assisted instruction compared to traditional bedside teaching | AI Tool (ChatGPT v4); Used to explore case vignettes with interactive feedback and educator review | Beneficial |
| Cheloff et al, 2021 [147] | Undergraduate; Medical students | To develop and pilot an electronic communication program with patients | Patient Portal; Used for forwarding patient messages to students for drafting and approval | Beneficial |
| Herrmann-Werner et al, 2021 [148] | Undergraduate; Second year medical students in communication course | To examine use of speech-to-speech translation app in training | Translation App; Used for taking histories from standardized patients with real-time translation | Inconclusive |
| Sun et al,  2020 [149] | Postgraduate; Medical interns | To evaluate new communication software | Communication Software; Used for online learning and communication assessment with SEGUE framework | Mixed |
| White et al, 2022 [150] | Postgraduate; Residents (years 1–4) in pathology, O&G, internal medicine | To evaluate video-based communication assessment as training | VCA Software; Used for specialty-specific video cases with feedback and replay | Beneficial |

**References for included studies are listed in the main manuscript reference list (see references [30–150]).*
