# Supplementary material for: Use of Digital Technology for Developing Communication Skills in Undergraduate and Postgraduate Medical Education: Scoping Review
Source: JMIR Med Educ. 2026 Apr 20;12:e87012. doi: 10.2196/87012 (PMC13094807; doi:10.2196/87012)

Figure 4. Distribution of studies examining digital communication skills training in medical education across all learners by technology type and year of publication.

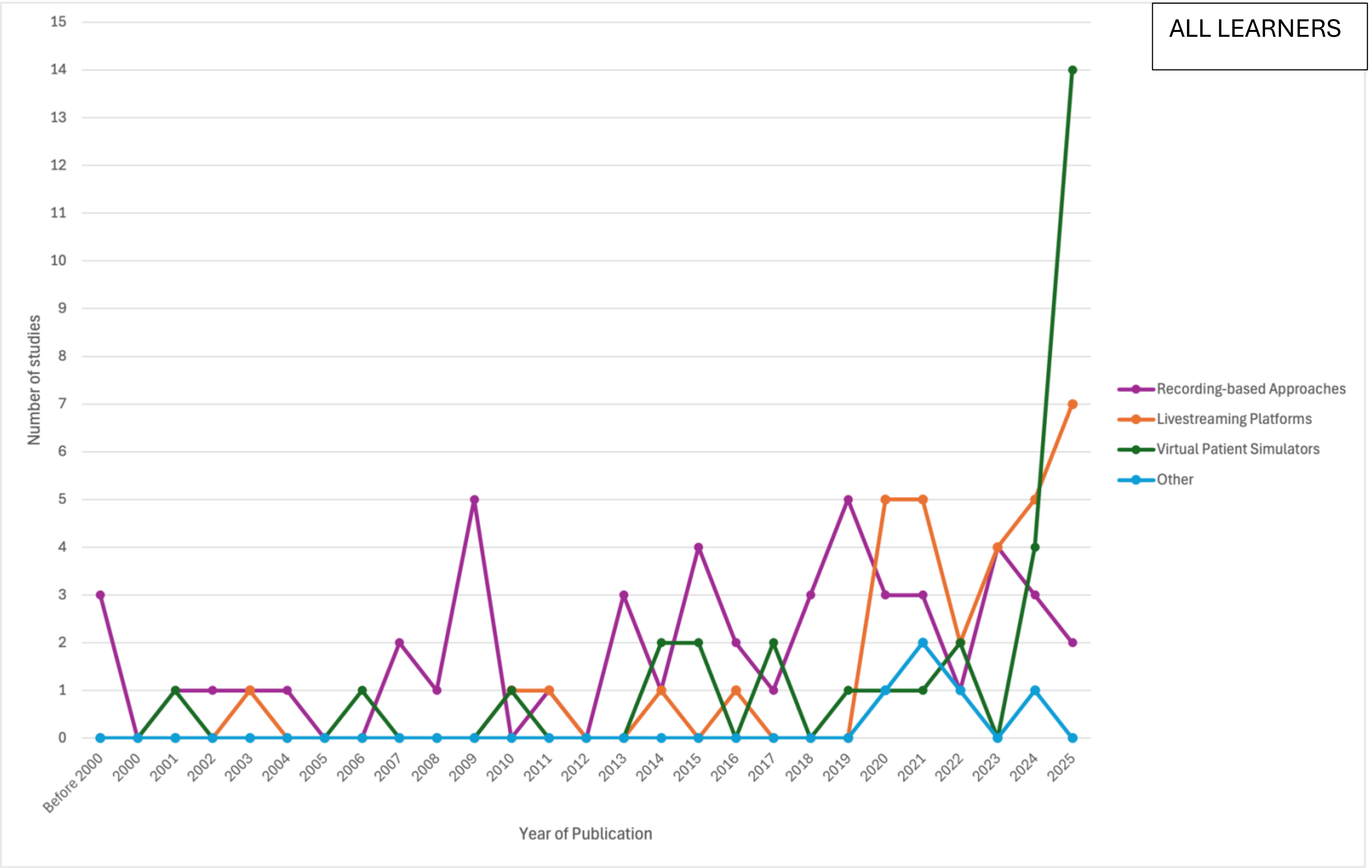

Figure 5. Distribution of studies examining digital communication skills training in medical education among undergraduate learners by technology type and year of publication.

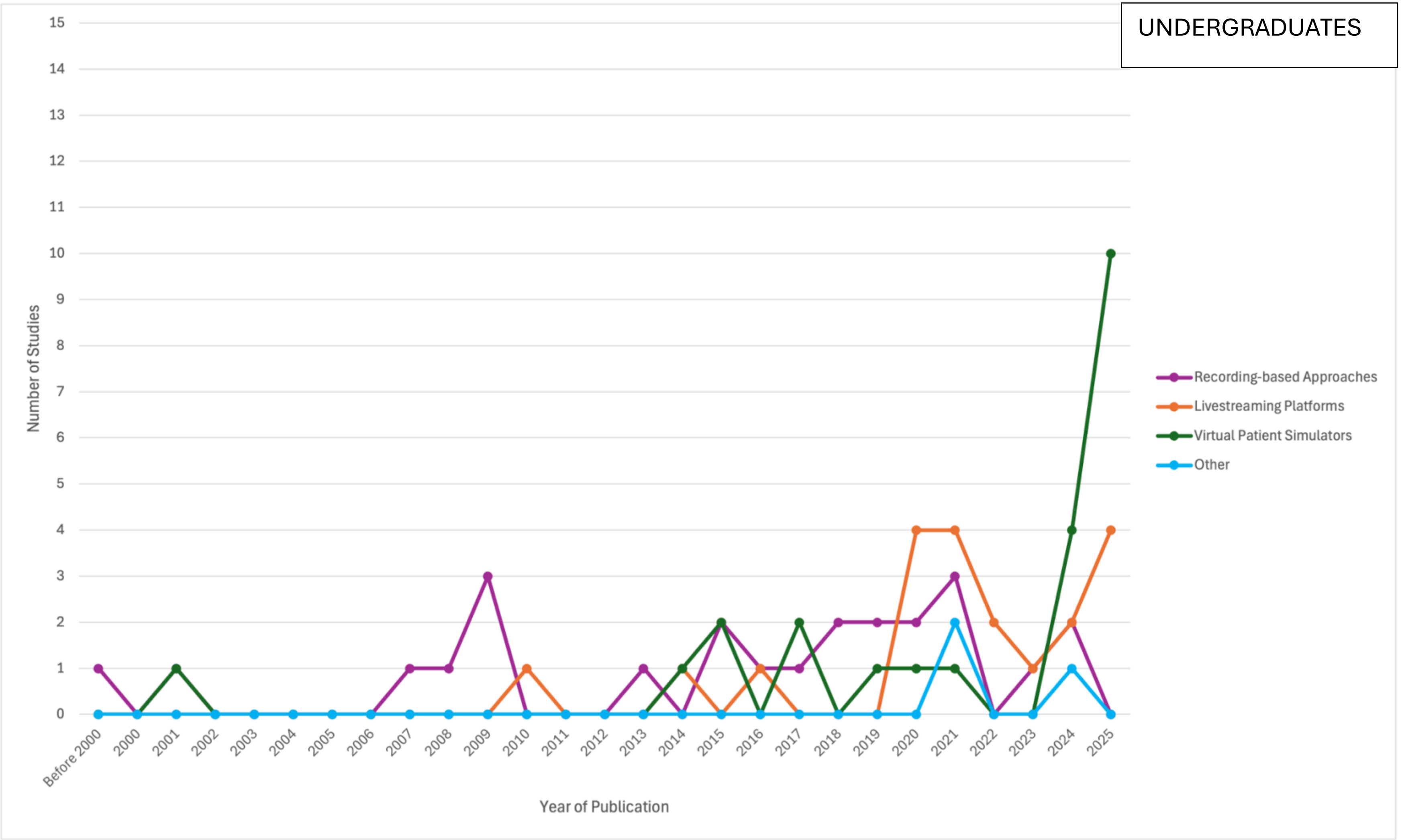

Figure 6. Distribution of studies examining digital communication skills training in medical education among postgraduate learners by technology type and year of publication.

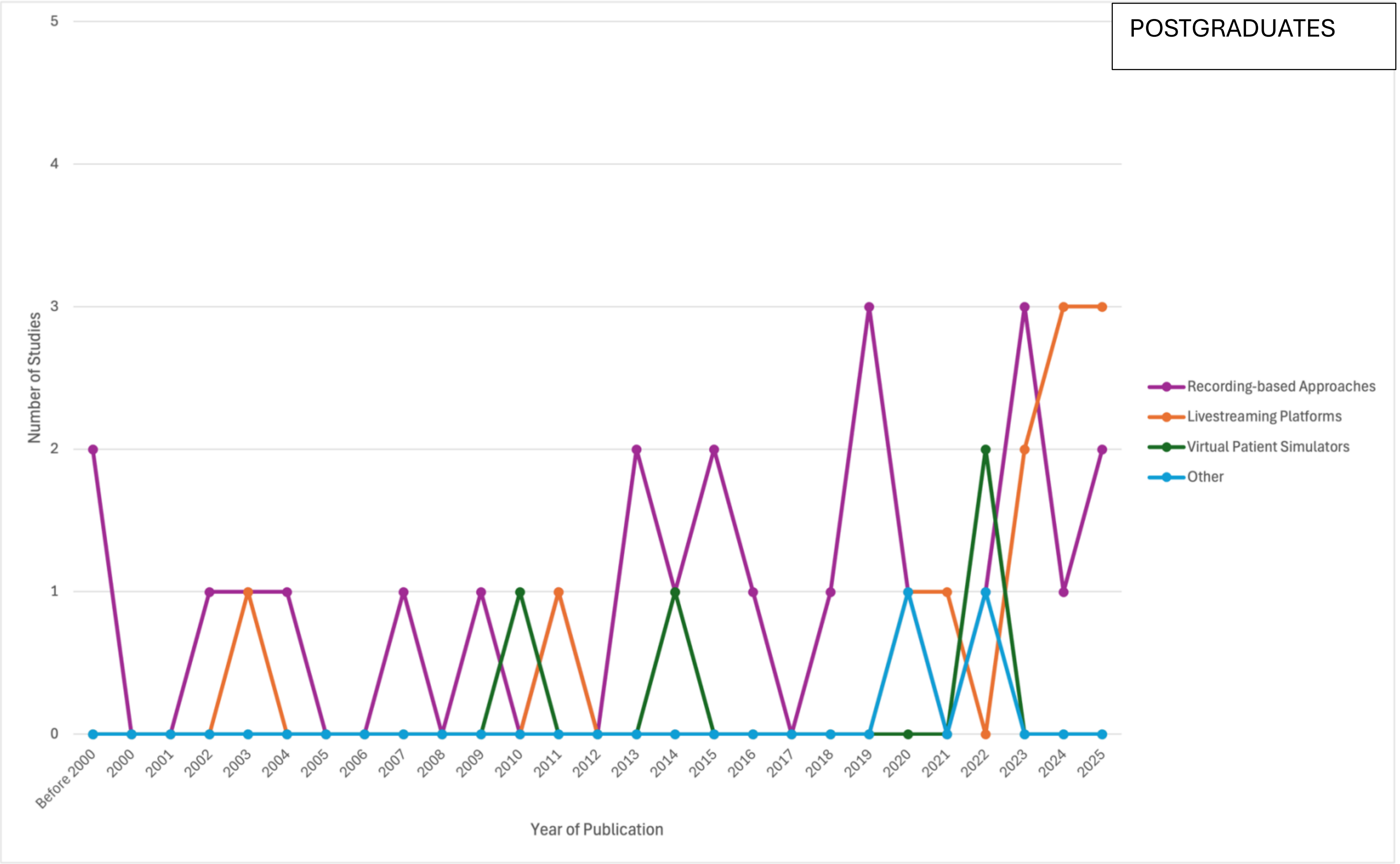

Supplement: Multimedia Appendix 4 [file mededu-v12-e87012-s004.pdf]
