# Supplementary figures and images for: Use of Digital Technology for Developing Communication Skills in Undergraduate and Postgraduate Medical Education: Scoping Review
Source: JMIR Med Educ. 2026 Apr 20;12:e87012. doi: 10.2196/87012 (PMC13094807; doi:10.2196/87012)

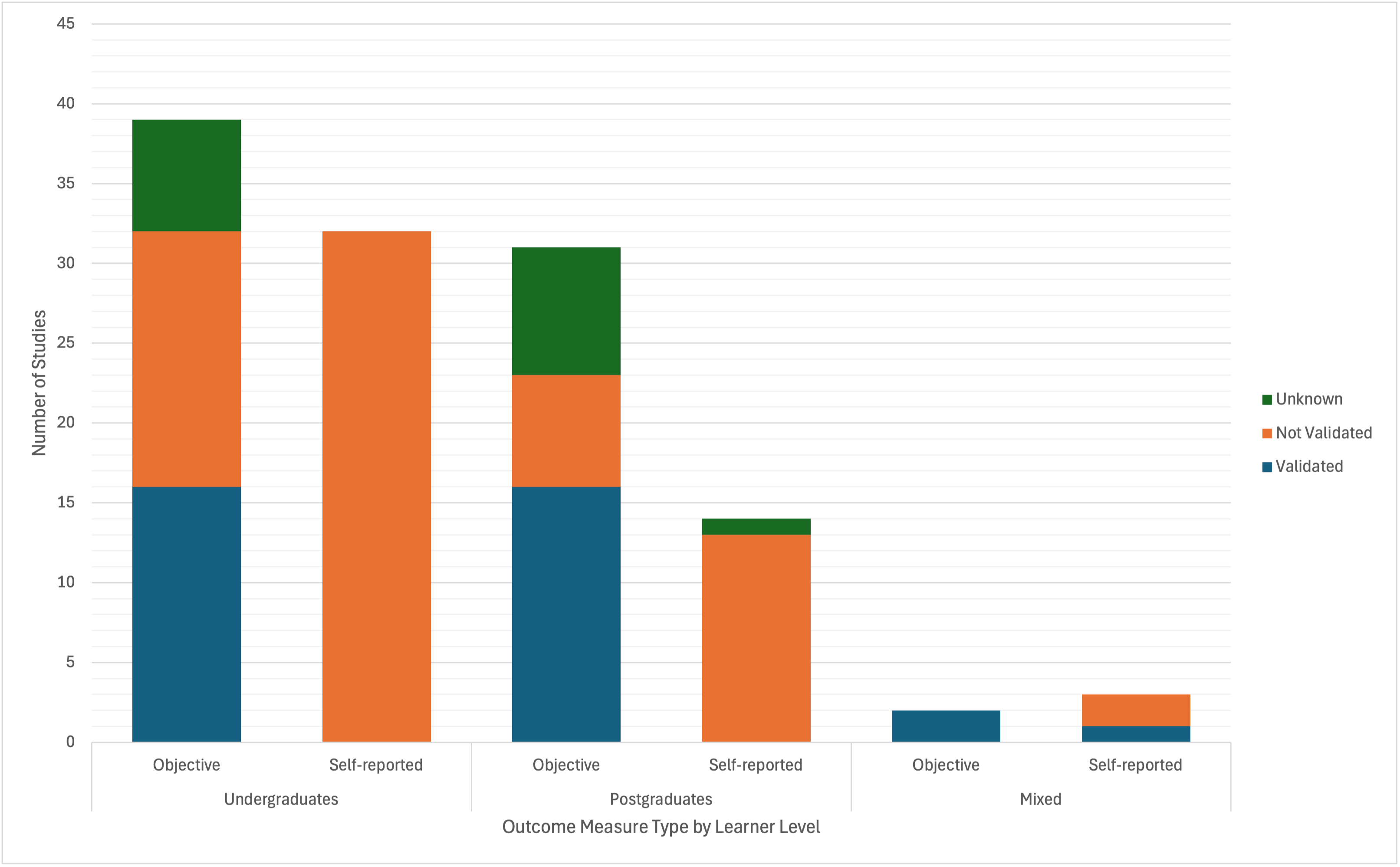

Supplement: Multimedia Appendix 5 [file mededu-v12-e87012-s005.png]
