# Supplementary material for: Use of Digital Technology for Developing Communication Skills in Undergraduate and Postgraduate Medical Education: Scoping Review
Source: JMIR Med Educ. 2026 Apr 20;12:e87012. doi: 10.2196/87012 (PMC13094807; doi:10.2196/87012)
Supplement: Multimedia Appendix 6 [file mededu-v12-e87012-s006.pdf]

Multimedia Appendix 8.

Figure 8. Distribution of studies examining digital communication skills training in medical education across Kolb's experiential learning stages by technology type.

Technology Type	Kolb's Experiential Learning Cycle				
	Stage 1: Concrete Experience	Stage 1 & 2	Stage 2: Reflective Observation	Stage 3: Abstract Conceptualisation	Stage 4: Active Experimentation
Recording-based Approaches	◐	●	●	○	○
Livestreaming Platforms	●	●	◐	○	○
Virtual Patient Simulators	●	◐	◐	◐	○
Other	◐	◐	○	○	○

Legend:

● Substantial evidence (n≥10) ◐ Limited evidence (n=1-9) ○ No evidence (n=0)

Figure 9. Distribution of studies examining digital communication skills training in medical education across Kirkpatrick's evaluation levels by technology type.

Technology Type	Kirkpatrick's Level of Evaluation			
	Level 1: Reaction	Level 2: Learning	Level 3: Behavior	Level 4: Results
Recording-based Approaches	◐	●	◐	◐
Livestreaming Platforms	◐	●	◐	○
Virtual Patient Simulators	◐	●	○	○
Other	◐	○	○	○

Legend:
● Substantial evidence (n≥10) ◐ Limited evidence (n=1-9) ○ No evidence (n=0)
