# Supplementary material for: Use of Digital Technology for Developing Communication Skills in Undergraduate and Postgraduate Medical Education: Scoping Review
Source: JMIR Med Educ. 2026 Apr 20;12:e87012. doi: 10.2196/87012 (PMC13094807; doi:10.2196/87012)
Supplement: Multimedia Appendix 7 [file mededu-v12-e87012-s007.pdf]

Multimedia Appendix 9. Evidence map of studies examining digital communication skills training in medical education (N=122).

Reference	Study Design							Learner Level			Communication Skills Focus						Kolb's Learning Stage				Kirkpatrick's Evaluation Level				Outcome Measures			
	Randomised Controlled Trials	Quasi-Experimental	Cross-sectional Study	Non-randomised Comparative	Mixed Methods	Qualitative Study	Descriptive Case Study	Undergraduate	Postgraduate	Mixed	General Communication & History Taking	Difficult Conversations (BBN[a], GOC[b], DOME[c])	Communication with Defined Patient Groups	Shared Decision-Making & Consent	Telecommunication / Remote Consultations	Patient Education & Information Giving	Stage 1: Concrete Experience	Stage 2: Reflective Observation	Stage 3: Abstract Conceptualization	Stage 4: Active Experimentation	Level 1: Reaction	Level 2: Learning	Level 3: Behaviour	Level 4: Results	Validated Objective	Non-validated Objective	Validated Self-reported	Non-validated Self-reported
Recording-based Approaches (n=51)																												
Beyth et al. 2009 [30			x						x				x				x	x			x							x
Bonnaud-Antignac et al. 2010 [31]		x						x				x					x	x				x				x		
Bos-vanden Hoek et al, 2023 [32]		x							x					x			x	x				x			x			
Busenius et al, 2022 [33]				x				x			x						x					x			x			
Cals et al. 2007 [34]		x							x		x						x	x				x				x		

Carrard et al, 2020 [35]		x						x				x					x	x				x			x			
Denizon Arranz et al, 2021 [36]		x						x			x						x	x				x				x		
Dohms et al, 2020 [37]		x							x		x							x				x			x			
Dowling et al, 2007 [38]		x							x		x						x	x				x				x		
Evens et al. 1983 [39]		x							x		x						x	x				x						x
Farnill et al, 1997 [40]		x						x			x						x	x				x				x		
Fischbeck et al, 2020 [41]			x					x				x					x					x			x			
Freytag et al, 2022 [42]			x						x		x						x	x			x							x
Hardoff et al, 2016 [43]		x							x				x					x				x						x
Harendza et al, 2023 [45]			x					x			x						x	x				x			x			
Harnof et al, 2013 [44]			x						x					x				x				x						x
Hulsman et al, 2009 [46]			x					x			x							x				x						x
Hulsman et al, 2015 [47]			x					x			x							x				x				x		
Kaltman et al, 2018 [48]	x							x			x						x	x				x				x		
Knowles et al, 2001 [49]	x							x			x							x				x				x		
Kobayashi et al, 2023 [50]		x							x		x							x				x			x			
Liu et al, 2016 [51]	x							x			x						x	x				x			x			
Levine et al,	x								x				x					x					x					x

2025 [79]																												
Makaricheva et al, 2024 [52]		x						x			x							x				x				x		
Mauksch et al, 2013 [53]		x						x			x							x				x						x
Mohos et al, 2020 [54]			x					x			x						x	x			x							x
Moulton et al, 2009 [55]	x									x	x							x				x			x			
Muller et al, 2019 [56]				x					x					x			x	x				x			x			
Naik et al, 2018 [57]			x					x			x						x				x							x
Noordman et al, 2011 [58]			x						x		x							x			x					x		
Noordman et al, 2019 [59]		x							x		x							x				x			x			
Ohlmann et al, 2024 [60]			x					x								x		x				x				x		
Ozcakar et al, 2009 [61]	x							x			x							x				x				x		
Perron et al, 2015 [62]				x				x			x						x	x				x				x		
Pless et al, 2021 [63]			x					x			x							x				x						x
Ravitz et al, 2013 [64]		x							x				x					x				x			x			
Roter et al, 1995 [66]	x								x			x					x	x						x	x			
Roter et al, 2004 [65]		x							x				x					x				x			x			
Ruesseler et al, 2017 [67]				x				x						x				x					x		x			
Scardovi et al, 2003 [68]		x							x		x							x					x		x			

Setubal et al, 2018 [69]	x								x			x					x	x				x				x		
Slort et al, 2014 [70]	x								x			x					x	x				x			x			
Smith et al, 2002 [71]					x				x		x						x	x			x					x		
Smith et al, 2023 [72]		x							x			x					x	x				x			x			
Supiot et al, 2008 [73]		x						x				x					x	x				x			x			
Trent et al, 2015 [74]				x					x				x				x	x				x				x		
Van Rossem et al, 2019 [75]							x	x			x						x	x				x						x
White et al, 2024 [76]	x								x			x					x	x				x				x		
Xiao et al, 2025 [80]		x							x			x						x					x					x
Yuan et al, 2019 [77]		x							x			x					x	x				x			x			
Zick et al, 2007 [78]						x		x			x						x	x					x					x
Live-streaming Platforms (n=33)																												
Abraham et al, 2021 [81]			x					x			x						x				x							x
Afonso et al, 2020 [82]			x					x			x						x				x							x
Aluce et al, 2024 [83]		x							x			x					x	x					x			x		
Bittner et al, 2016 [84]				x				x								x	x	x				x				x		
Booth et al, 2022 [85]		x						x							x		x				x							x

Bramstedts et al, 2014 [86]			x					x					x				x					x						x
Clever et al, 2003 [87]			x						x					x			x	x				x				x		
Daetwyler et al, 2010 [88]	x							x				x					x					x			x			
Deming et al, 2024 [89]		x							x			x					x				x							x
Geng et al, 2025 [113]		x							x		x							x				x			x			
Godoy-Pozo et al, 2023 [90]		x						x			x						x	x				x						x
Gur et al, 2024 [91]				x				x					x				x	x				x						x
Hayes et al, 2025 [92]		x						x				x					x	x				x				x		
Heller et al, 2023 [93]			x						x						x		x	x				x				x		
Holmes et al, 2020 [94]			x						x			x					x	x			x							x
Iammeechai et al, 2025 [95]			x					x					x				x					x						x
Jones et al, 2025 [96]		x							x						x		x					x						x
Khawand-Azoulay et al, 2025 [97]			x					x				x					x	x				x						x
Knie et al, 2020 [98]			x					x								x	x	x				x						x
Lenes et al, 2020 [99]			x					x			x						x				x							x
Mack et al, 2025 [100]		x							x			x					x					x						x

Newcomb et al, 2021 [101]		x						x			x						x	x				x				x		
Newcomb et al, 2022 [102]		x						x			x						x	x				x			x			
Pang et al, 2021 [103]		x						x						x			x					x						x
Phillips et al, 2023 [104]		x							x		x						x				x					x		
Pozo et al, 2025 [105]			x					x							x		x	x				x						x
Rasalam et al, 2020 [106]			x					x			x						x	x			x							x
Rivet et al, 2023 [107]		x								x		x					x	x				x			x			
Ruddock et al, 2021 [108]			x					x					x				x	x				x						x
Sasnal et al, 2021 [109]			x						x		x						x				x							x
Taylor et al, 2024 [110]					x				x						x		x	x				x				x		
Tsui et al, 2024 [111]	x							x					x				x	x				x				x		
Yudkowsky et al, 2011 [112]		x							x		x						x	x				x				x		
Virtual Patient Simulators (n=32)																												
Andrade et al, 2010 [114]		x							x			x					x					x			x			
Bearman et al, 2001 [115]					x			x			x						x				x					x		
Borg et al, 2024 [116]				x				x			x						x				x							x
Bruen et al, 2017 [117]				x				x					x				x	x				x				x		
Carrard et al, 2020 [118]						x		x				x					x				x							x

Chiu et al, 2025 [137]		x						x				x						x				x				x		
Comulada et al, 2025 [138]		x						x			x							x				x						x
Courteille et al, 2014 [119]					x			x			x						x				x				x			
Davidovics et al, 2025 [139]					x			x			x							x				x						x
Detering et al, 2014 [120]		x							x			x								x		x				x		
Dickerson et al, 2006 [121]	x							x			x						x					x				x		
Foster et al, 2015 [122]	x							x				x					x	x				x				x		
Frey-Vogel et al, 2022 [123]		x							x							x	x				x							x
Herschbach et al, 2025 [140]		x							x		x							x			x							x
Jacklin et al, 2021 [124]					x			x						x			x					x						x
Jadoon et al, 2025 [121]					x					x	x							x				x						x
Kleinsmith et al, 2015 [125]				x				x			x						x					x			x			
Kron et al, 2017 [126]	x							x				x					x	x				x				x		
Lee et al, 2025 [142]	x							x			x							x				x			x			
McCarrick et al, 2025 [127]	x							x			x						x					x			x			
McCarthy et al, 2022 [128]		x							x							x	x					x			x			
Mool et al, 2024 [129]					x			x			x						x					x						x

Mukadam et al, 2025 [130]		x						x				x					x	x				x						x
Poulose et al, 2025 [131]			x							x	x						x	x				x						x
Raafat et al, 2024 [132]		x						x			x						x					x				x		
Sezer et al, 2019 [133]	x							x			x						x	x				x				x		
Suarez-Garcia et al, 2025 [143]		x						x					x					x				x			x			
Thompson et al, 2025 [134]		x								x			x				x					x					x	
Tyrell et al, 2025 [144]		x						x			x							x				x						x
Wang et al, 2025 [135]	x							x			x						x					x				x		
Yamamoto et al, 2024 [136]		x						x			x						x	x				x			x			
Young et al, 2025 [145]		x						x			x							x				x						x
Other (n=5)																												
Ba et al, 2024 [146]	x							x			x						x					x			x			
Cheloff et al, 2021 [147]			x					x			x						x				x							x
Herrmann-Werner et al, 2021 [148]			x					x					x				x				x							x
Sun et al, 2020 [149]		x							x		x						x					x			x			
White et al, 2022 [150]		x							x			x					x	x				x				x		

[a]BBN: Breaking Bad News; [b]GOC: Goals of Care; [c]DOME: Disclosure of Medical Error

***References for included studies are listed in the main manuscript reference list (see references [30–150]).**
